# From Nature to Innovation: Exploring Natural Biopolymers in 3D Bioprinting for Bone Regeneration

**DOI:** 10.1021/acsomega.6c01732

**Published:** 2026-06-03

**Authors:** Rafaella Moreno Barros, Antônia Carla de Jesus Oliveira, Luíse Lopes Chaves, Amanda Damasceno Leão, Renata Kelly Luna Gomes Ramos, Gabrielle de Lima Maniçoba, Fábio Rocha Formiga, Mônica Felts de La Roca Soares, José Lamartine Soares Sobrinho

**Affiliations:** † National Institute of Science and Technology of the Economic Industrial Health Complex, Department of Pharmaceutical Sciences, 28116 Federal University of Pernambuco-UFPE, Av. Prof Artur de Sá, S/N − Cidade Universitária, Recife, Pernambuco 50740-520, Brazil; ‡ Capibaribe Laboratory, Department of Pharmaceutical Sciences, Federal University of Pernambuco-UFPE, Av. Prof Artur de Sá, S/N − Cidade Universitária, Recife, Pernambuco 50740-520, Brazil; § Aggeu Magalhães Institute, Oswaldo Cruz Foundation - FIOCRUZ, Department of Immunology, UFPE campus, Cidade Universitária, Recife, Pernambuco 50670-420, Brazil; ∥ Graduate Program in Applied Cellular and Molecular Biology, Institute of Biological Sciences, University of Pernambuco-UPE, Recife, Pernambuco 50100-130, Brazil

## Abstract

Critical bone defects represent a global health challenge, necessitating specialized medical care and imposing significant costs on the public systems. In this context, many efforts in the tissue engineering and regenerative medicine framework have been made, focusing on developing biomaterials for bone regeneration. Additive manufacturing, known as 3D printing, has emerged as a promising technology for creating revolutionary biomedical devices. An advancement of this technique, 3D bioprinting, enables the fabrication of biomaterials intended for tissue regeneration and the transplantation of synthetic organs, using bioinks (cell-enriched hydrogels) and inks (hydrogels with or without bioactive agents) to produce a variety of devices, such as scaffolds, dressings, microneedles, and blood vessels. Thus, biopolymers have emerged as promising materials for the development of biomaterials and bioinks in tissue engineering because of their biocompatibility, biodegradability, low toxicity, and ability to mimic the extracellular matrix. Therefore, this review discusses recent advances in natural biopolymer-based bioinks for 3D bioprinting in bone regeneration, highlighting rheological properties, mechanical performance, biological functionality, and strategies such as chemical modification, nanomaterials, and smart systems. In addition, challenges related to vascularization, structural stability, immune responses, and clinical application are discussed.

## Graphical abstract

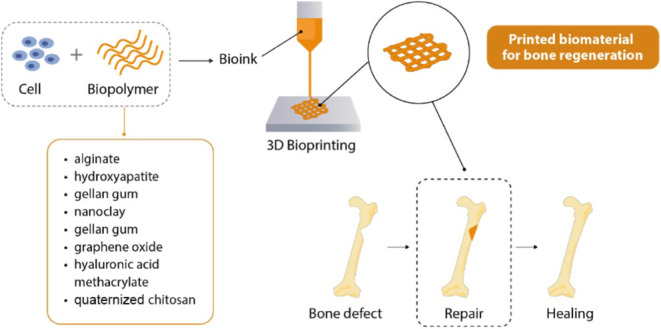

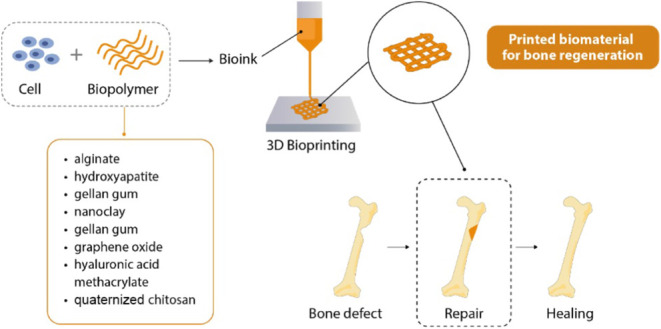

## 1 Introduction

Bone tissue has the ability to self-regenerate, allowing bones to rebuild themselves in shape, size, and strength, thus returning to their original characteristics.^1^ However, in critical size bone defects (CSBD), which are characterized by a defect length greater than 1–2 cm and a loss of greater than 50% of the bone circumference, healing is a complex process, as endogenous mechanisms are compromised, especially local revascularization, which can result in loss of function and permanent damage to the limb.^2^ The 2019 Global Burden of Disease, Injuries, and Risk Factors Study estimated that there are 178 million new cases of bone fractures globally each year, driving the search for specialized treatment and new technologies in orthopedics.^3^ Currently, traditional therapeutic interventions are based on autograft, allograft, xenograft, or synthetic biomaterials and have limitations such as availability of donor tissue, greater probability of rejection, and postoperative complications.^4^

In recent years, advances in science in tissue engineering and regenerative medicine (TERM) have positively impacted the acquisition of new biomaterials and technologies, which aim to contribute to the development of alternative therapies, from studies at the molecular level to the development of functional organs and tissues.^5^ Thus, one of the technologies used at TERM is additive manufacturing (AM), or 3D printing, which deposits material layer by layer, through computer-aided design (CAD), to obtain prints with high precision, resolution, and reproducibility.^6^ This innovation has led to the emergence of 3D bioprinting, in which it is possible to print materials containing cells, growth factors, and bioactive molecules. In this way, highly organized 3D tissues and organs can be obtained with controlled spatial orientation, which offer appropriate cellular environments and replicate natural characteristics and functions.^7^

However, to ensure the success of 3D bioprinting, in addition to equipment, adequate laboratories, and human resources, it is necessary to adopt biocompatible raw materials with mechanical characteristics similar to the natural extracellular matrix (ECM), which are important aspects for cell adhesion, dissemination, migration, proliferation, and differentiation.^8^ 3D bioprinting technology uses materials such as ceramics, metals, composites, synthetic polymers, and biopolymers, which enables the development of inks and bioinks for biomedical applications.^9^ Unlike inks, bioinks contain, in addition to the hydrogel, cells or growth factors. In order to optimize the 3D bioprinting process, the appropriate bioink must present a fast sol–gel transition, which reduces processing time. To ensure high shape fidelity and structural stability, chemical (e.g., glutaraldehyde, genipin, UV irradiation with photoinitiators) and physical (e.g., pH, temperature, ions, hydrophobic interactions) cross-linking can be used.^10^

The bioinks used in 3D bioprinting for bone regeneration can be derived from natural and synthetic biopolymers, individually or in combination. Natural biopolymers (e.g., alginate, collagen, gums, and chitosan) interact with the cells and proteins of the ECM, promoting cell adhesion and differentiation.^11,12^ On the other hand, synthetic biopolymers (e.g., poly­(ε-caprolactone) (PCL), poly­(ethylene glycol) (PEG), and polycaprolactone (PCL)) offer control over the degradation rate and mechanical properties.^13,14^ They are biocompatible, biodegradable, have the ability to form three-dimensional networks, and offer a biomimetic matrix that provides cellular activity and bone neoformation.^15^ An important characteristic of biopolymers is the possibility of performing chemical modifications (e.g., quaternization, methacrylation, oxidation) and physical modifications (e.g., variation of pH, temperature, concentration) to develop material with better viscosity, rheology, biocompatibility, and degradation rate conditions necessary to print stable and functional 3D scaffolds for bone regeneration.^16^

Nevertheless, cell viability, integration with natural bone, and optimization of the mechanical properties of bioinks are major challenges. Thus, other innovative technologies such as smart bioinks and 4D printing are promising candidates in the development of new biomaterials for bone regeneration.^17^ In this context, the objective of this review is to explore the use of natural biopolymers in the development of inks and bioinks for 3D bioprinting applied in bone regeneration, as well as to present studies on recent applications, challenges, and future perspectives for the improvement of these biomaterials, aiming at their clinical application in personalized bone regeneration.

## 2 Bone Tissue and Critical Bone Defect

Bone tissue is a mineralized and vascularized connective tissue that forms the vertebrate skeleton, accounting for approximately 15% of body weight. It serves multiple functions, including providing mechanical support, protecting organs, and enabling movement through interaction with the musculoskeletal system. Additionally, it possesses self-regenerative capacity. Structurally, it is composed of cortical (compact) bone, responsible for mechanical strength, and trabecular (spongy) bone, which is highly porous (≈80–90%) and contains bone marrow, along with the periosteum.^18–20^ It is formed by specialized and progenitor cells, primarily during embryonic development, with cranial bone originating from both the neural crest and the mesoderm. Its stiffness arises from a collagen-based extracellular matrix mineralized with calcium phosphate, predominantly hydroxyapatite (Ca_10_(PO_4_)_6_(OH)_2_).^21,22^ This hierarchical organization of bone tissue across multiple length scales is illustrated in Figure 1.

**Figure 1:**
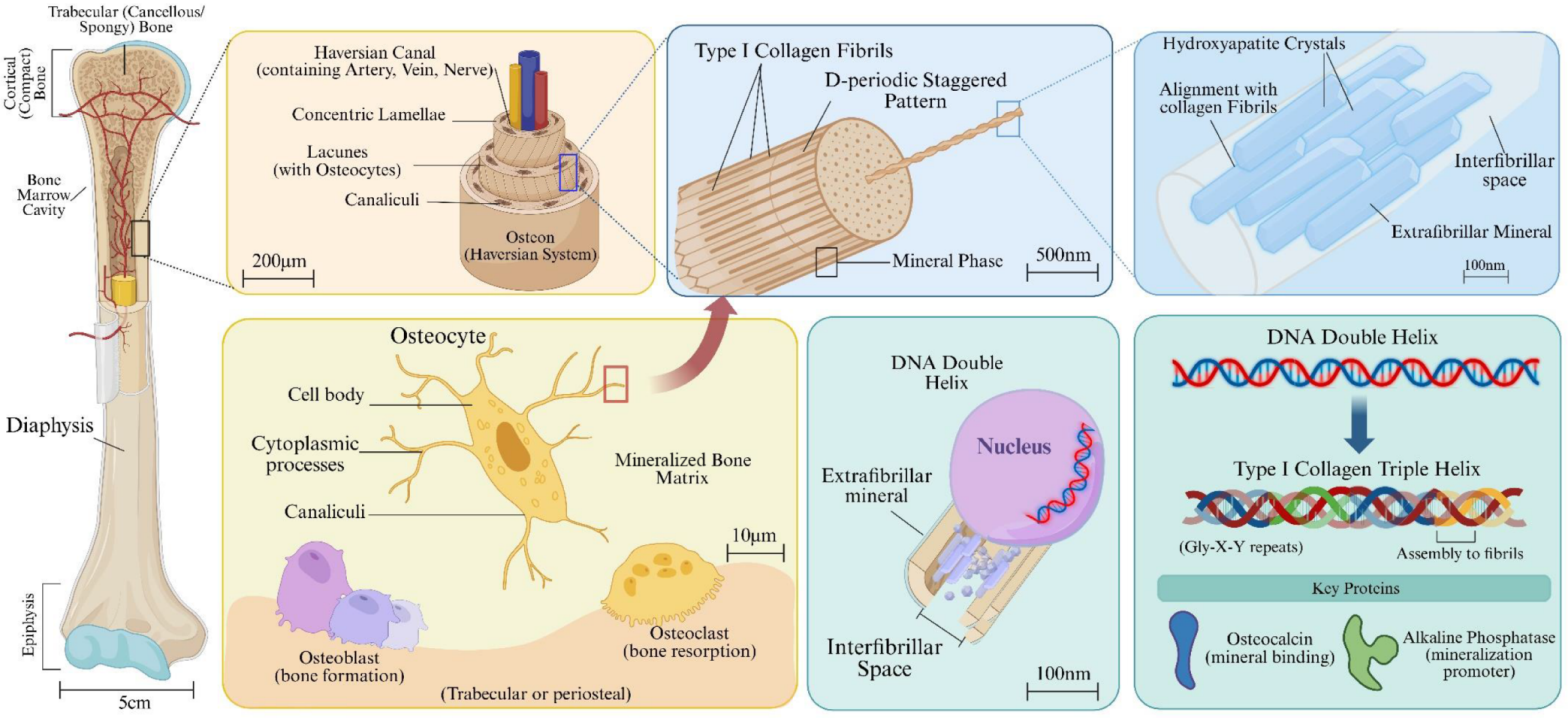
Multiscale hierarchical organization of bone tissue, from macroscopic structure to collagen fibril mineralization with hydroxyapatite, highlighting osteonal architecture, cellular components, and key features of collagen assembly and biomineralization.

This complex organ performs essential biological functions, including mineral storage, calcium homeostasis, blood pH regulation, immune cell differentiation, and hematopoiesis. Bone marrow is responsible for hematopoietic activity, producing blood cells and maintaining stem cells that are critical for the circulatory and immune systems. It comprises osteoblasts, osteocytes, osteoclasts, bone marrow-derived mesenchymal stem cells (BMSCs), adipocytes, and vascular endothelial cells. Furthermore, it is classified into red and yellow types, as summarized in Figure 2, along with their respective locations and functions.^23–25^

**Figure 2:**
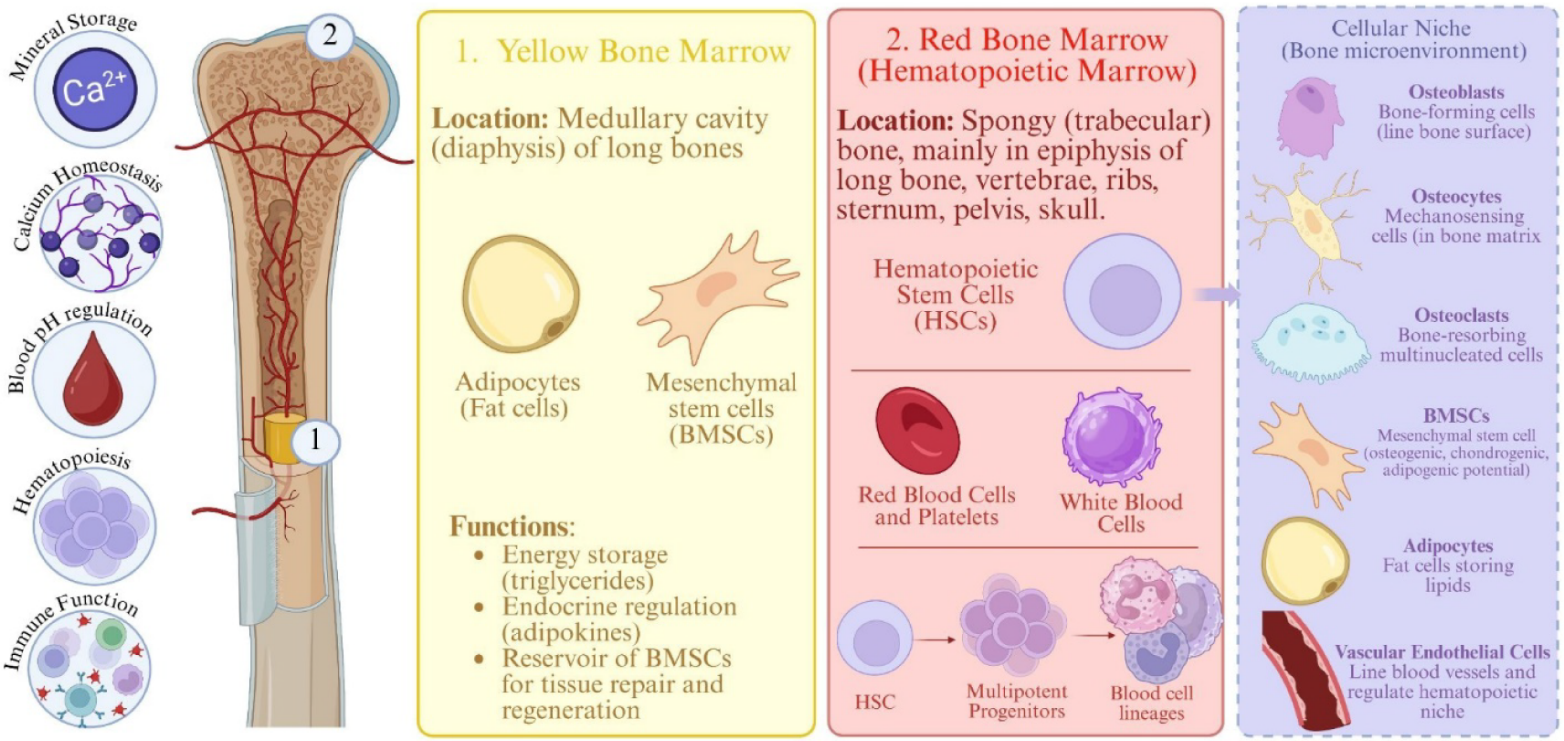
Bone marrow compartments and functions. Yellow marrow is associated with energy storage and mesenchymal stem cells, while red marrow supports hematopoiesis. The bone marrow niche comprises multiple cell types that regulate bone remodeling and blood cell formation.

Despite its regenerative capacity, conditions such as osteogenesis imperfecta, osteoporosis, and trauma can lead to critical bone defects (CBDs), impairing self-repair.^1^ CBDs are generally defined as defects >1–2 cm or involving >50% of bone circumference, with severity influenced by anatomical location and surrounding tissues.^26^ They are classified as cavitary (minimal biomechanical impact but affecting fixation) or segmental (compromising structural stability).^27^ Treatment is further challenged by patient-related and clinical factors, often resulting in increased morbidity, intervention rates, and healthcare costs.^28^

Bone nonunion, defined as the failure of fracture healing within 6–9 months, remains a major challenge in bone regeneration. It arises from mechanical factors (e.g., instability, inadequate immobilization, bone loss) and biological factors (e.g., poor vascularization, infection, diabetes, osteoporosis).^29^ With an incidence of up to 10%, treatment often requires surgical intervention and depends on both mechanical and biological conditions, as described by the “diamond concept,” which includes osteogenic cells, osteoconductive scaffolds, growth factors, mechanical stability, and vascularization.^30–32^ However, no current substitute integrates all these components, highlighting the need for advanced biomaterials for effective bone regeneration.^33^

Conventional bone repair includes biological grafts (autograft, allograft, xenograft) and synthetic biomaterials. Autografts remain the “gold standard” due to their osteoconductive, osteoinductive, and osteogenic properties, while minimizing immune rejection and disease transmission. However, their use is limited by donor site morbidity, the need for additional surgery, and restricted availability for large defects.^34,35^ Allografts are more readily available but show limited bone ingrowth (<5 mm after two years) and present risks such as antigenicity, immune rejection, disease transmission, abnormal remodeling, and poor integration.^36,37^ Xenografts carry similar risks and require thermal sterilization to reduce prion transmission, which can compromise osteoinductivity and alter scaffold structure.^36,38^ Due to poor integration and high rejection rates, their use in orthopedics has largely been discontinued.^39^

The limitations of these procedures, combined with the global prevalence of bone defects, highlight the need for novel bone biomaterials. However, key factors regulating bone regeneration remain incompletely understood, limiting the development of optimal solutions.^40–42^ Biomaterials primarily interact with surrounding tissue through osteoconduction and osteoinduction, while composition, structure, surface morphology, and additives further influence regenerative outcomes.^41,43^

To address these challenges, tissue engineering has emerged as a multidisciplinary field focused on developing biomaterials for tissue repair and regeneration.^44,45^ To achieve this, it integrates principles from biology, chemistry, and physics and is based on three key components, namely, cells, scaffolds, and growth factors.^46^ Given the limited availability of tissues for transplantation, tissue engineering is considered a promising alternative, particularly with advances such as 3D bioprinting, which enables the fabrication of precise and functional scaffolds.^47,48^

## 3 3D Bioprinting as an Innovative Tool in Bone Regeneration

Additive manufacturing, or 3D printing and 3D bioprinting, is an innovative approach to developing biomaterials for bone and other tissue regeneration, as it allows the creation and printing of customized scaffolds specific to each patient. It is considered a rapid prototyping technique that was described in 1986 by Charles W. Hull. Supported by specialized software, such as computer-aided design (CAD), biomaterials are deposited layer by layer in predefined positions, creating porous structures with complex geometries and tunable bioactivity for different biomedical applications.^49^ Compared to traditional techniques, such as synthetic grafts and cell therapy, 3D bioprinting has advantages and some limitations.^50^

Unlike synthetic grafts for bone regeneration, such as hydroxyapatite and calcium phosphate, which have the ability to mimic the mineral structure of bone, 3D bioprinting allows the creation of scaffolds with personalized, biocompatible architecture, incorporation of cells, growth factors, and bioactive molecules. It offers advantages such as greater precision in controlling the geometry, mechanical properties, and degradation of the scaffold, in addition to promoting a microenvironment more favorable to bone regeneration.^51,52^ Moreover, synthetic grafts have limitations in integration with natural bone tissue, healing, and biological response, as they do not favor the regeneration of bone cells efficiently and may result in rejection or reabsorption of the graft over time.^52^

Other strategies such as cell therapy use mesenchymal stem cells (MSCs) or autologous osteoblasts, which have the ability to form new bone tissue and promote healing. The main limitations are the need for large-scale cell culture, the risk of tumor formation, the complexity of obtaining and applying the cells, and the lack of a support structure to promote cell adhesion, proliferation, and differentiation, which makes it difficult to repair large bone defects.^53^ In addition, there are technical and clinical challenges, such as process complexity, equipment, specialized materials, human resources, high cost, and others.^54^ Thus, 3D bioprinting has become fundamental in tissue engineering, especially in the research and development of bone substitutes, where the complexity of the structure and functionality of bone tissue presents significant challenges.^55^

### 3.1 Bioprinting Techniques

The standardization organizations ASTM and ISO categorize the AM process into seven different types: powder bed fusion (PBF), material extrusion (ME), vat photopolymerization (VP), material jetting (MJ), binder jetting (BJ), sheet lamination (SL), and directed energy deposition.^56^ Although ASTM/ISO classifies additive manufacturing into seven distinct categories, techniques based on extrusion, material jetting, and vat photopolymerization stand out as the most suitable for applications in tissue engineering, especially in the production of polymeric bioinks. This is mainly due to these methods’ ability to process polymeric materials and hydrogels under mild temperature and pressure conditions, preserving the cellular viability and biological activity of the incorporated components. Other categories such as powder bed fusion (PBF), directed energy deposition, and sheet lamination generally involve high temperatures, intense thermal energy, or conditions incompatible with biological systems, limiting their direct application in biofabrication.^56,57^

3D bioprinting can be classified into two main methods: scaffold-free bioprinting and scaffold-based bioprinting. In the first case, aggregates of living cells act as building blocks, printed directly to form three-dimensional tissues without the need for supporting matrices.^58^ In the scaffold-based technique, cells are encapsulated in polymer matrices called bioinks, which are printed in predefined shapes to generate support structures that facilitate cell adhesion and proliferation.^59^ In summary, bioprinting technologies can be categorized into three distinct classifications: extrusion-based, inkjet bioprinting, and laser-assisted and vat-based polymerization bioprinting,^15^ which have established themselves as the most promising approaches for the development of biomimetic scaffolds and constructs in tissue engineering. A schematic view of the bioprinting modalities discussed in this section is shown in Figure 3.

**Figure 3:**
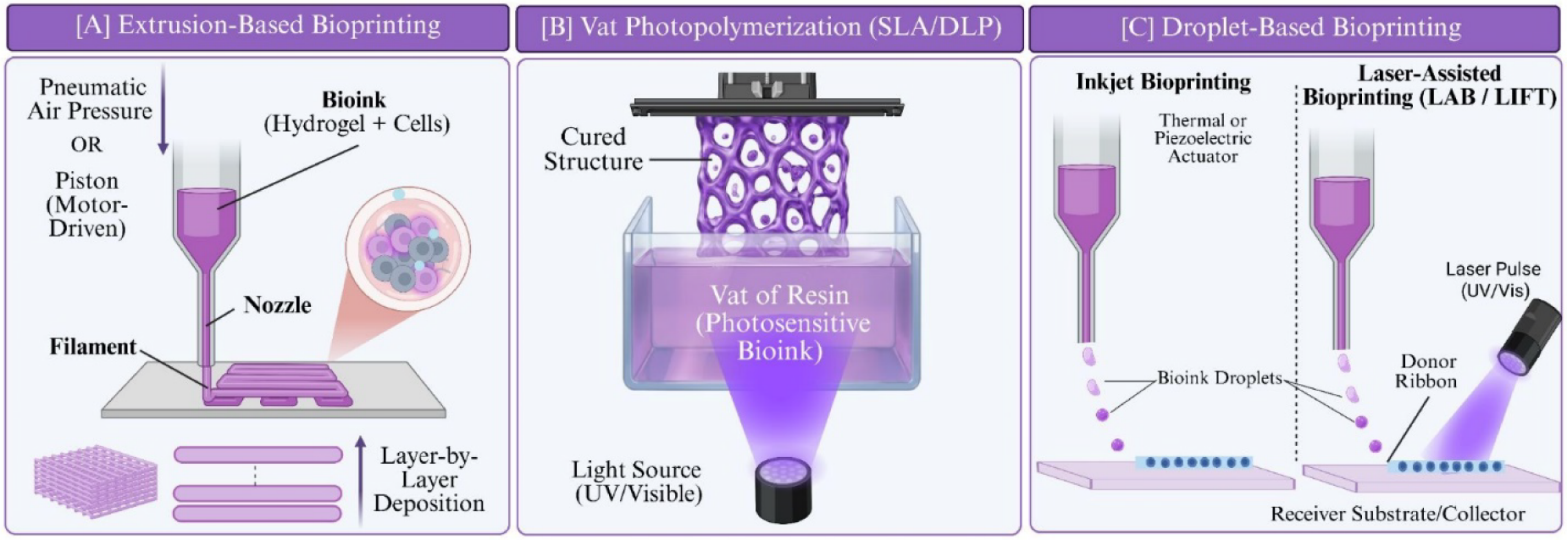
Schematic representation of major bioprinting modalities. (A) Extrusion-based bioprinting, where bioinks are deposited through pneumatic or mechanical extrusion in a layer-by-layer manner. (B) Vat photopolymerization (SLA/DLP), where photosensitive bioinks are selectively cured using a light source to form 3D structures. (C) Droplet-based bioprinting, including inkjet (thermal or piezoelectric actuation) and laser-assisted (LAB/LIFT) techniques, where discrete bioink droplets are deposited onto a substrate.

#### 3.1.1 Extrusion-Based Bioprinting

Extrusion-based bioprinting remains the most widely adopted technique for bone tissue engineering applications because of its versatility, cost-effectiveness, and compatibility with high-viscosity bioinks containing cells and bioactive factors. These techniques include pneumatic, piston, and screw bioprinting with two or more printheads capable of extruding bioink in continuous filaments through a small or even micro nozzle.^60^

Hydrogel-based bioinks have the capacity to create three-dimensional structures in a layer-by-layer manner. Cross-linking, which can be of an ionic, thermal, enzymatic, or photoinduced nature, can occur during or immediately following deposition in order to ensure structural integrity.^61^ The production of cell-laden constructs with a controlled architecture has been successfully achieved through the printing process. This method has been employed in the fabrication of scaffolds incorporating mesenchymal stem cells (MSCs), osteoblasts, and endothelial cells.^62^

Multimaterial extrusion systems have been used to create spatially arranged structures that resemble the hierarchical structure of natural bone, including distinct cortical and trabecular regions.^63^ It is a simple and reliable technique that involves a wide range of bioprintable biomaterials and use of affordable equipment. On the other hand, this approach is associated with deformation of the hydrogel, a relatively low resolution, potential clogging of the nozzle, and apoptosis of the embedded cells, mainly due to the induced pressure exerted inside the nozzle.^15,61^ For a material to be considered printable within the context of this technique, the 3D structure of the printed material must be preserved. The printability of extrusion bioinks is primarily determined by their rheological properties. It is essential that bioinks exhibit shear-thinning behavior, thereby facilitating extrusion under controlled shear stress while enabling rapid recovery of viscosity after deposition to maintain shape fidelity.^62^ These properties directly influence filament formation, layer stacking, and the geometric accuracy of the printed construct. Of particular significance is the necessity of meticulous optimization of the balance between viscosity and extrusion pressure, given the potential of excessive shear forces during printing to impede cell viability.

The evaluation of this criterion, specifically shear-thinning behavior and yield stress, is based on three separate but interconnected parameters: extrudability, filament fidelity, and structural integrity.^60^ The kinetics of cross-linking are equally critical, as they influence the degree of strand fusion and the overall stability of the printed construct. The incorporation of ceramic particles (e.g., hydroxyapatite or β-tricalcium phosphate) to promote bone regeneration has been shown to increase bioink viscosity and introduce flow disturbances. This necessitates close monitoring of particle size and concentration to prevent nozzle clogging.^62^

In parallel, cell survival is acutely sensitive to the mechanical forces applied during the process. It has been demonstrated that elevated extrusion pressures and flow rates result in a substantial augmentation of the shear stress experienced by cells as they traverse the nozzle. The effect is exacerbated by smaller nozzle diameters, which raise shear rates and increase the cell membrane stress. Additionally, prolonged printing times can lead to nutrient deprivation within the construct prior to perfusion, while excessive temperatures used in the extrusion of thermoplastic components can be cytotoxic. The selection and concentration of cross-linking agents must also be considered, as both rapid and chemically aggressive cross-linking processes have been observed to induce cytotoxic effects.^62,64^ Recent advances in extrusion bioprinting include the development of coaxial nozzle systems that can deposit multiple materials simultaneously, forming core–shell structures that improve mechanical properties while preserving cell viability.^65,66^

#### 3.1.2 VAT Photopolymerization: Stereolithography (SLA) and Digital Light Processing (DLP)

Vat photopolymerization (VAT/VPP), as defined by the ASTM and ISO, is the overarching term for any three-dimensional printing process that employs a vat of liquid photopolymer resin, which is selectively cured by a light source (typically UV).^67^ This concept can be divided into two primary categories: Stereolithography (SLA) was the first patented VAT technology, and Digital Light Processing (DLP) followed. In the standard SLA process, a point-source laser is utilized to delineate the cross-section of each layer, thereby “drawing” the object into the resin. Conversely, DLP employs a digital projector screen (typically a DMD chip) to flash an image of the entire layer in a single action, thus achieving a substantial increase in printing speed in comparison to the laser-tracing method employed in SLA.^68,69^

It has been demonstrated that SLA and DLP techniques offer superior resolution (10–100 μm) and printing speed in comparison to extrusion-based methods. This facilitates the fabrication of scaffolds with intricate microarchitectures that closely replicate the complex geometry of native bone tissue, with exceptional resolution and speed.^70^− However, it is important to note that the UV light exposure required for the polymerization process may result in cell damage during the bioprinting process.^15,63^

Recent studies have demonstrated the application of SLA and DLP for creating bone scaffolds with precisely controlled pore sizes, interconnectivity, and surface topography that promote osteogenic differentiation and bone ingrowth.^72^ DLP and other VP bioprinting techniques have been demonstrated to achieve micron-scale resolution, thus enabling the fabrication of complex biomimetic scaffold architectures, including gyroid, lattice, perfusable channels, and hierarchical structures for bone and cartilage. High-resolution light-based methods are utilized to replicate intricate tissue microarchitectures and discerning features that are paramount for bone and osteochondral units.^73^ These methods accentuate multiscale, hierarchical structures, and porosity. Photo-cross-linkable biomaterials and light-based bioprinting demand fast cross-linking, high resolution, and meticulous control of pore networks and microenvironments, rendering them well-suited for biomimetic bone scaffolds.^74–76^ Furthermore, VP technique has the capacity to fabricate cm-scale living constructs within seconds to minutes. This explicitly addresses the traditional speed–resolution trade-off and scalability issues.^77,78^

The utilization of photopolymers in VP is of particular significance, as it employs the photopolymerization-induced cross-linking and hardening mechanisms of photocurable materials.^56^ Biofabrication technologies based on VP are facilitated by the directing of a light beam onto the photoreactive precursor solution, thereby allowing the polymerization process to be precise and controlled over time. The printability in light-based systems is primarily determined by the photo-cross-linking kinetics and the light penetration depth, which are influenced by the wavelength and intensity of the light source.^62,70^ Different photoinitiators, such as Irgacure 2959, lithium phenyl-2,4,6-trimethylbenzoylphosphinate (LAP), eosin Y, and VA-086, are employed to initiate cross-linking reactions, thereby enabling the fabrication of complex, high-resolution tissue constructs while maintaining adequate cytocompatibility.^79,80^

Residual unreacted monomers or postprocessing solvents must be thoroughly removed, as they can cause long-term cytotoxicity. The presence of ceramic fillers may also necessitate higher light doses for adequate curing, indirectly increasing the risk of photodamage to encapsulated cells.^70,71^ Radical photoinitiators remain the most prevalent in bone-tissue-engineering applications. Irgacure 2959 (I-2959) and lithium phenyl 2,4,6-trimethylbenzoylphosphinate (LAP) are frequently applied in GelMA-, PEG-, PVA-, and chitosan-based systems for bone applications.^81,82^ I-2959 is a widely employed agent because of its low level of toxicity and compatibility with biomedical hydrogels. However, it exhibits limited aqueous solubility and typically necessitates UV radiation, which can elevate the risk of DNA damage and cell death if parameters such as dose and duration are not meticulously regulated.^81,83^

In contrast, LAP exhibits high water solubility, facilitating expedited and effective gelation at reduced concentrations. Additionally, it can be activated by both UV and longer-wavelength visible light, rendering it a highly attractive candidate for 3D bioprinting and injectable hydrogels intended for bone regeneration. However, its utilization is associated with elevated levels of reactive oxygen species generation, which may potentially compromise viability.^81–83^ Recent reviews have emphasized the development of “green” photoinitiators as promising alternatives to reduce systemic toxicity and regulatory restrictions.^82,84^ However, challenges remain regarding stability, degradation byproducts, and standardization of curing parameters in translational bone regeneration settings.

#### 3.1.3 Material Jetting and Laser-Assisted Bioprinting

Inkjet bioprinting operates by ejecting discrete bioink droplets onto a substrate through thermal or piezoelectric actuation.^71^ Unlike the extrusion method, inkjet bioprinters are designed to work with low-viscosity bioinks. The inkjet bioprinting method can be categorized into two distinct approaches: continuous bioprinting and drop-on-demand (DoD). In the latter, single droplets are deposited according to a predetermined path. The DoD technique is based on three different droplet generation mechanisms: piezoelectric, thermal, and electrostatic.^85^

The laser-assisted bioprinters (Laboratories) consist of the laser source, the target, and the receiver plate. One of the most common laser-assisted bioprinter techniques is known as laser-induced forward transfer (LIFT).^15^ LIFT uses high-energy laser pulses to create a bubble in a donor ribbon, propelling cell-laden material onto a receiver substrate without the need for a nozzle.^85^ The process involves the application of a laser pulse to a thin film of bioink that absorbs the laser energy and subsequently generates a bubble. The expansion of the bubble creates a high-speed jet of the liquid bioink. Upon contact with the substrate, this jet gives rise to fine droplets of the liquid. Subsequent to this initial process, the laser is repositioned to another location on the surface of the bioink, and the process is repeated to create a pattern or a structure of interest.^86,87^ Compared to other techniques, LIFT enables high cellular densities and viability, with fine printing resolution. It also accommodates a wider range of viscosities compared with other drop-on-demand bioprinting techniques. Besides, the nozzle-free printing method eliminates clogging problems.^15,87^

In the context of inkjet systems, printability is constrained by narrow windows of bioink viscosity and surface tension, which are required for stable droplet formation and flight. It has been demonstrated that elevated levels of cell concentrations or particulate loads can increase the risk of nozzle clogging and disrupt the droplet consistency. In the LAB, printability is governed by laser fluence, pulse duration, and the properties of the donor layer. Collectively, these factors determine the fidelity of the transferred droplets. The wettability of the receiver substrate has been demonstrated to have a significant impact on the processes of droplet spreading and fusion.^64,71^

With regard to cell viability, it has been demonstrated that the transient heat pulses employed for droplet generation in thermal inkjet printing can result in cellular damage if the peak temperature or duration exceeds the cellular tolerance threshold. Piezoelectric systems, while capable of avoiding thermal stress, have the potential to induce mechanical damage through high-frequency pressure transients and shear forces.^62,64,71^ In LAB methods, excessive laser may result in both local heating and mechanical shock. Furthermore, the impact stress upon landing on the substrate has been shown to cause cell injury, particularly at high droplet velocities. The incorporation of protective components within the bioink, such as extracellular matrix elements or specific osmolarity adjusters, has been demonstrated to facilitate the modulation of these mechanical stresses.^64^

### 3.2 Biomaterial Scaffolds for Bone Tissue Engineering

3D bioprinted biomaterials are classified as “Third Generation Biomaterials” because they are bioactive and biodegradable. They also have the characteristic of being biomimetic (bio = life; mimesis = imitation), a term that refers to materials, structures, or processes that seek to imitate the properties, functions, and mechanisms of nature.^88,89^ They are designed to replicate the biological and functional characteristics of natural tissue, promoting controlled interaction with biological tissues and providing support for cell growth and tissue integration.^7^ Furthermore, they exhibit advanced functional properties, such as controlled degradation, stimulation of specific cellular responses, and molecular interaction with the biological microenvironment.^90^

To achieve ideal bone regeneration conditions, the biomaterials used must present biomimetic properties, which mimic the characteristics and functions of natural bone tissue, as well as have favorable mechanical properties, controlled biodegradability, compatibility with clinical sterilization processes, and the ability to be visualized in imaging exams without compromising structural information.^91^

#### 3.2.1 Ideal Criteria for Bioinks

The successful application of bioinks in BTE is critically dependent on the integration of physicochemical, biological, and processing-related properties. Collectively, these properties ensure printability, structural integrity, and biological functionality.^83,92,93^ An ideal bioink must exhibit two fundamental qualities: a high biocompatibility and cytocompatibility. It should support cell adhesion, proliferation, and osteogenic differentiation, while also being free of any toxicity arising from raw materials, degradation byproducts, or cross-linking agents. Furthermore, minimal immunogenicity is imperative to prevent adverse inflammatory responses and to promote tissue integration after implantation.^83,92,93^

The mechanical properties of bioinks must be tailored to approximate those of the target bone tissue, particularly in terms of compressive strength and stiffness, to provide an initial structural support and maintain scaffold integrity during the early stages of regeneration. However, given the inherent mechanical limitations of numerous hydrogel-based systems, the implementation of reinforcement strategies, such as the incorporation of ceramic phases or polymer blending, is frequently necessary to improve load-bearing capacity.^83,92^

Furthermore, the biofunctionalization of materials with growth factors and bioactive particles, including hydroxyapatite, bioactive glasses, and calcium silicate cements, has been demonstrated to possess the capacity to enhance the osteoconductivity. These components play a central role in promoting osteoinduction, osteoconduction, and vascularization, which are fundamental processes for effective bone regeneration. Conversely, these additives have the capacity to modify local pH, ionic release, and the inflammatory response, necessitating meticulous balancing.^94,95^

Cross-linking mechanisms are of particular significance in determining the stability and performance of the printed construct. It is fundamental to meticulously select physical, chemical, or photoinduced cross-linking strategies to ensure expeditious stabilization without compromising cell viability. The kinetics and conditions of cross-linking have a direct impact on the mechanical strength, degradation rate, and biological response of the scaffold. It is essential to emphasize that the degradation profile of the bioink must be meticulously designed to align with the rate of new tissue formation.^83,92,93^ The ideal bioink must be capable of producing a scaffold that degrades in a controlled manner. This is to ensure that mechanical support is maintained during the early healing stages while being gradually replaced by newly formed bone tissue. The degradation products must be nontoxic and should not interfere with cellular activity or local physiological conditions.^83,92^

#### 3.2.2 Biomaterials-Based Scaffolds

The requirements for bioinks and 3D-printed scaffolds are not identical but are interdependent. While bioinks must demonstrate appropriate rheology, controlled gelation, high cell viability during extrusion, and high printability and geometric fidelity, the resulting scaffolds are governed by additional and more stringent criteria regarding structural and functional performance in the medium and long term.^76,96^

In the context of scaffolds derived from biomaterials, the primary focus lies on the mechanical properties, hierarchical porous architecture, and controlled degradation. The objective is to align the modulus and strength with the demands of bone (including under load), to ensure sufficient porosity and interconnectivity for osteogenesis, angiogenesis, and nutrient diffusion, and to coordinate the resorption of the material with the formation of new bone tissue.^76,97,98^

Scaffolds should allow cell attachment, proliferation, and differentiation while being noncytotoxic and eliciting minimal immune response. The biocompatibility of a biomaterial or biomedical device can be defined as its ability to perform its function while maintaining an appropriate host response within a specific application. It can be determined by observing the extent of adverse changes in the host response that affect the homeostasis following implantation. Thus, in BTE, scaffolds should allow cell-to-cell communication via biomolecular signaling while being nontoxic to the surrounding host tissue.^99^ The main classes of biomaterials used include metals, ceramics, synthetic and natural polymers, in addition to composites, which are used in the formulation of hydrogels, nanofiber scaffolds, and 3D bioprinted biomaterials.^41^

Several bioactive materials have been studied and applied in the development of bioprinted biomaterials for bone regeneration. Among them, bioactive bioceramics, such as bioactive glass and hydroxyapatite, stand out; polymer-ceramic composites, such as polylactide (PLA);^100^ biodegradable bioactive polymer matrices, such as polycaprolactone (PCL), polyglycolide (PGA), and poly­(D,l-lactide-*co*-glycolide) (PLGA);^101^ bioactive hydrogels; collagen-based biomaterials;^102^ and nanomaterials and nanocomposites, such as carbon nanotubes and bioactive polymeric nanofibers, have been explored.^103^ Furthermore, 3D bioprinted scaffolds can be engineered to release growth factors (GFs) required for bone regeneration. Numerous studies have reported the role of different GFs and have presented the potential applications in bone healing and osteogenesis for regulating cell behavior, including recruitment, migration, adhesion, proliferation, and differentiation, such as platelet-derived growth factors (PDGFs), bone morphogenic proteins (BMPs), insulin-like growth factors (IGFs), transforming growth factors (TGFs-ß), and vascular endothelial growth factors (VEGFs).^104^

3D scaffolds are used in bone regeneration and in the fixation of prostheses through osseointegration. They are generally formed with composite ECM and a complex noncellular structural network, responsible for assisting in cell binding for cell differentiation, growth control, and tissue formation. The properties of 3D scaffolds can be controlled, including surface area, interconnectivity between pores, permeability, and mechanical strength.^46^

Bone tissue architecture is a critical feature in BTE, as structure has an impact on both mechanical properties and biological response. The porous architecture of BTE scaffolds is commonly influenced by physical attributes such as porosity, pore size, surface area, and interconnectivity. Porosity refers to the volume percent of free space in the framework, while pore size is the average size of pores in the scaffold. Scaffold surface area is a function of pore sizes present on the surface and scaffold porosity. The scaffold interconnectivity is a function of the pores’ spatial location and their size and porosity, which determines the extent of cell infiltration and nutrient transport.^105^

In order to promote cell adhesion and osteogenesis, bioprinted biomaterials must have sufficient porosity to allow nutrient penetration, tissue vascularization to allow migration and proliferation of active cells, and, consequently, the formation of new mineralized bone tissue. It has been reported that suitable porosities for scaffolds used for BTE applications range from 50% to 80%, depending on the required mechanical performance.^105^ ^,^ Regarding pore size, pores greater than 300 μm are accepted to facilitate new bone formation and vascularization, while the minimum accepted size is around 100 μm. Porosity, in turn, can affect the mechanical properties of the scaffold and, consequently, its degradation rate. Scaffold porosity should be well controlled and aligned with the scaffold life cycle, dictating the rate of fluid ingress as it degrades and maintains its load-bearing capacity.^105^

In addition to the pore size and density, pore arrangement and scaffold geometry can affect bone healing and its ability to support vascular growth, nutrient diffusion, and mechanical properties. The scaffold geometry commonly observed in the BTE can be divided into randomized and regular structures. Randomized orientations are those ones that lack any repeating units, tending to mimic the natural bone morphology. On the other hand, regular orientation structures include cubic, hexagonal, triply periodic minimal surfaces (TPMS), spherical, and honeycomb arrangements.

The surface roughness of the scaffold directly affects the ability of host cells to attach and modulates the crosstalk between cells involved in bone regeneration depending on the cell type. For example, fibroblasts appear to adhere better to smooth surfaces, whereas they are stimulated to proliferate and synthesize collagen when seeded on more uniform surfaces. In contrast, epithelial cells should adhere better to rough surfaces.^99^ Therefore, an optimal design should provide sufficient surface area for 3D scaffolds to support neovascularization and bone integration, while facilitating oxygen and nutrient diffusion, as well as the expulsion of waste products.^106^

Another important factor is the choice of bioprinting technology, as it directly impacts the quality of the scaffolds produced. For example, 3D scaffolds with a nanoporous structure, modified surface, and different chemical compositions can modulate the immune response and promote osteogenesis by creating an immunomodulatory microenvironment.^107^ According to Wang et al.,^8^ 3D bioprinting using a coaxial nozzle has been used to manufacture scaffolds that favor vascularization and cell growth. However, the channels formed are small and static, measuring around 100 μm, which hinders cell migration and the development of an immunomodulatory microenvironment necessary for bone regeneration. Also, parallel channels are inflexible and static, which makes it difficult for cells or blood vessels to migrate quickly into the channels. These challenges are currently being explored to optimize bioprinting in bone tissue engineering.^108^

### 3.3 Biological and Immunological Aspects of Biopolymaterial Bioinks

In addition to the raw materials adopted in the biomaterial, the repair of bone defects requires favorable immunomodulation. The immune system identifies natural biomaterials as foreign bodies, activates immune cells (macrophages, dendritic cells, and T cells), and, consequently, releases proinflammatory cytokines and chemokines, which attract other immune cells to the implantation site.^109^ Depending on the characteristics of the biomaterial and immune response, this process can trigger a favorable or harmful inflammatory response. However, biomaterials can modulate the behavior of immune cells and influence the immune response.^110^

Recent advances in hydrogel-based systems for cancer immunotherapy have demonstrated the critical role of biomaterials in modulating the local immune microenvironment. In particular, hydrogels have been engineered to respond to specific physicochemical cues and regulate immune cell behavior within the tumor microenvironment, thereby enhancing therapeutic outcomes. Interestingly, similar challenges are observed in bone tissue engineering, where the immune response plays a pivotal role in determining the regeneration success. Therefore, strategies developed to modulate the immunosuppressive tumor microenvironment may offer valuable insights for the rational design of bioinks and scaffolds aimed at promoting proregenerative immune responses in bone healing.^111,112^

Thus, biopolymeric bioinks have effects on the biological and immunological responses of bone regeneration, as they interact directly with the immune system, influence inflammatory responses, promote tissue regeneration, and, in some cases, can trigger adverse immune reactions.^112,113^ This behavior is closely related to the ability of hydrogels to mimic the extracellular matrix and support cell adhesion and proliferation.^114^ The selection of polymeric components is a critical step in the development of these materials, as both natural and synthetic biomaterials can significantly influence the host immune response, while the tunable physicochemical properties of hydrogels allow their design to be tailored for specific biomedical applications. Natural biopolymers are often associated with favorable biocompatibility; however, assuming that they inherently exhibit low inflammatory potential is an oversimplified and not always accurate interpretation.^114,115^

Furthermore, the immunological behavior of these materials is influenced by several factors, including their source, degree of purity, molecular structure, cross-linking method, degradation byproducts, sterilization process, and the presence of residual contaminants such as endotoxins. Therefore, many biological responses attributed to the polymer itself may, in fact, be more closely related to the material processing and preparation steps than to its intrinsic properties.^116–118^

Macrophages are central regulators of the response to biomaterials due to their marked phenotypic plasticity. When activated, M1-type macrophages secrete proinflammatory mediators, including tumor necrosis factor-alpha (TNF-α), interleukin-1β (IL-1β), and interleukin-6 (IL-6), contributing to the initial antimicrobial defense and the clearance of cellular debris.^119,120^ In contrast, activated M2-type macrophages release anti-inflammatory and reparative factors, such as interleukin-10 (IL-10), transforming growth factor-β (TGF-β), and vascular endothelial growth factor (VEGF), thereby promoting angiogenesis, matrix remodeling, and tissue repair.^120,121^

It is important to note that the M1/M2 classification represents a simplified continuous spectrum rather than a rigid binary classification and that macrophage states in vivo are often more heterogeneous.^122^ However, the proper transition from the initial inflammatory phase to a proregenerative profile is generally associated with improved biomaterial integration and more efficient bone repair.^123^

During bone regeneration, signals released by macrophages directly promote the recruitment of mesenchymal stem cells, stimulate osteogenic differentiation, support blood vessel formation, and contribute to the functional balance between osteoblasts and osteoclasts.^124,125^ In this context, hydrogels have emerged as key platforms in tissue engineering, as they provide a supportive microenvironment that enhances cell proliferation, differentiation, and extracellular matrix formation.^114^

Natural biopolymers can actively participate in these immunological processes, particularly by influencing macrophage polarization, cytokine release, and the progression of the host response to the presence of foreign bodies.^126,127^ Therefore, understanding how specific polymers, such as alginate, chitosan, and collagen, modulate these pathways is essential for the rational development of next-generation immunomodulatory bioinks.^128^

Alginate is one of the most widely used polysaccharides in hydrogels and bioinks due to its mild ionic gelation and tunable physicochemical properties.^129^ In its purified form, alginate is often considered to be relatively bioinert. However, inflammatory responses may arise from residual impurities, endotoxin contamination, or byproducts generated during ionic cross-linking. Its biological behavior is further influenced by the mannuronic/guluronic acid ratio, matrix stiffness, degradation profile, and chemical functionalization.^130,131^ Nonetheless, comparisons among studies remain limited by the lack of standardization in alginate characterization, including purity, endotoxin burden, M/G ratio, and cross-linking conditions. This variability makes it difficult to distinguish whether the observed immunomodulatory effects arise from the polymer itself or from factors associated with its modification and processing.^132,133^

Chitosan is a cationic polysaccharide with antimicrobial properties, biodegradability, and the ability to modulate innate immune responses. Due to its positive charge, chitosan can interact with cell membranes and pattern-recognition receptors, often inducing an initial M1 macrophage response characterized by increased secretion of TNF-α, IL-6, and nitric oxide, partly mediated by Toll-like receptor signaling.^134^ Although this proinflammatory response may be beneficial during the early stages of healing or in contaminated defects, excessive or prolonged activation can lead to chronic inflammation and fibrotic tissue formation.^135^ It is important to emphasize that the immunological behavior of chitosan strongly depends on its degree of deacetylation, molecular weight, charge density, solubility, degradation products, and formulation strategy.^136^ Strategies such as chemical modification, blending with other polymers, and the incorporation of bioactive molecules have been investigated to reduce excessive inflammation and encourage a shift toward proregenerative macrophage phenotypes. However, systematic studies correlating the physicochemical properties of chitosan with long-term in vivo bone healing outcomes are still scarce.^137^

Collagen, the main component of the extracellular matrix, is widely used due to its intrinsic bioactivity and structural similarity to native tissues. Its domains containing the RGD (Arginine–Glycine–Aspartic acid) sequence promote cell adhesion and tissue colonization, making collagen particularly attractive for regenerative applications.^138,139^ Collagen-based biomaterials are frequently associated with a reduced foreign body response and enhanced M2-type macrophage polarization. Nevertheless, these effects are not universal, since the immunological behavior of collagen depends on factors such as its source, purification method, the presence of residual telopeptides, xenogeneic contaminants, sterilization procedures, scaffold architecture, and degradation kinetics.^140,141^ Moreover, cross-linking strategies introduced to improve mechanical stability may alter ligand accessibility, stiffness, and cell–matrix interactions, thereby reshaping macrophage responses.^142^ Recent engineered collagen membranes, including asymmetric or Janus-type architectures, have demonstrated the ability to modulate local immunity while promoting osteogenesis.^143^ Therefore, collagen should not be considered intrinsically immunomodulatory, but rather a biologically active platform whose host response depends on its source, processing, and structural engineering.^144^

Collectively, alginate, chitosan, and collagen demonstrate how different biopolymers can provide complementary immunological functions. Alginate offers tunable matrix properties and can be tailored to promote anti-inflammatory responses; chitosan may provide an initial stimulatory signal when properly controlled; and collagen creates a cell-responsive microenvironment that supports tissue regeneration.^145–147^ Together, these characteristics suggest that composite systems may offer advantages over single-component materials by coordinating sequential immune events, ranging from early defense and debris clearance to inflammation resolution, vascularization, and new bone formation.^148^ ^,^ From a translational perspective, relatively few studies have integrated these materials through a rational design that considers the spatial and temporal roles of each component. Most reports still evaluate isolated formulations under simplified in vitro conditions, whereas clinical healing involves dynamic, complex, and patient-specific immune responses.^149^

Future progress will likely depend on the development of “smart” biomaterials capable of responding to local stimuli, such as pH changes, enzymatic activity, or hypoxia, and releasing immunomodulatory signals, including cytokines, exosomes, or bioactive ions, in a controlled manner.^114,150^ In parallel, advanced physicochemical characterization techniques—including Fourier-transform infrared spectroscopy (FTIR), X-ray diffraction (XRD), Raman spectroscopy, and thermal analysis—may help establish correlations between material structure, processing parameters, and biological performance. This integrated approach can accelerate the rational design and optimization of biomaterials by enabling a more comprehensive understanding of biomaterial–host interactions.^114^

In the case of bone regeneration, biopolymeric bioinks interact with osteoblasts and other cells involved in the regeneration process. This facilitates the adhesion, proliferation, and differentiation of these cells into bone tissue.^151^ It is worth noting that bioinks containing osteoinductive factors, such as bone morphogenetic proteins (BMPs) or signaling molecules, can promote osteogenesis and accelerate the healing of bone defects.^152^ Bioinks can be developed to release growth factors in a sustained and controlled manner, making the microenvironment favorable for bone regeneration (e.g., thermoresponsive nanocomposite bioink).^153^ Furthermore, undesirable immune responses can be minimized, and osteogenesis can be improved through the strategy of functionalizing biopolymers with bioactive molecules, encapsulating MSCs, or immunomodulatory agents. Therefore, this process will optimize the interaction between the bioink and the surrounding tissue, while modulating the local immune response to prevent inflammation and promote tissue integration.^110,154^

The development of bioinks focusing on cellular interactions, immune modulation, osteoinduction, and advances in 3D bioprinting technology are important aspects to consider when seeking to develop new biomaterials to treat bone defects and injuries.^155^ Modulating the immune response ensures that 3D bioprinted scaffolds have the ability to regenerate bone tissue without causing rejection or excessive inflammation. To ensure the efficacy and biocompatibility of these biomaterials, one challenge is to stimulate the immune response with a balance between tolerance and immune activation.^156^

Immunological tolerance is when the immune system recognizes the biomaterial as compatible and does not generate an excessive inflammatory response, which favors the integration of the biomaterial and bone regeneration.^156^ The main challenge is not to completely suppress the immune response but rather to direct its progression from an initial controlled inflammatory phase toward resolution, vascularization, osteogenesis, and stable tissue integration.^157^ On the other hand, excessive or prolonged immune activation may lead to chronic inflammation, fibrotic encapsulation, or implant failure, ultimately compromising the bone regeneration. Therefore, the biological and immunological properties of biopolymeric bioinks are fundamental to their regenerative performance and clinical potential in bone tissue engineering.^148,158^

## 4 Biopolymers for 3D Bioprinting: Classification, Structure, and Chemical Modification

Biopolymers are biological macromolecules with either a linear or branched structure, whose characteristics can be modified by altering, for example, the chemical structure of the monosaccharide units or the degree of polymerization. Thus, they perform a wide range of functions in living organisms, being a versatile raw material.^159^

Because they are considered biocompatible and have a customizable chemical structure, biopolymers can be used in the development of health products, such as biomaterials (dressings for burns, bone scaffolds, heart valves, etc.),^160^ biomedical devices (equipment coating),^92^ nanotechnology (active pharmaceutical ingredient delivery systems),^161^ and 3D bioprinting (bioinks).^162^ Currently, in the field of bone regeneration, this natural raw material is being used in 3D bioprinting and in the composition of formulations (inks and bioinks) to obtain bone substitutes, membranes, scaffolds, dressings, etc.^163^ Its biodegradability, biocompatibility, and structural similarity to the extracellular matrix favor its application in the field of bone regeneration, providing important properties for an integral tissue formation, in addition to reducing the need for postsurgical procedures.^164^

A systematic classification of biopolymers can be achieved according to three criteria: their origin, chemical composition, and functional characteristics. It is evident that, on the basis of their origin, these substances can be broadly categorized into two distinct groups: natural biopolymers and synthetic polymers of biological relevance.^165^ Natural biopolymers are obtained from renewable biological sources, such as plants, animals, and microorganisms, whereas synthetic polymers may be chemically engineered to mimic biological structures or derived from biobased monomers.^166^

Within the field of natural biopolymers, notable examples include proteins (such as collagen, silk, fibrinogen, and others) and polysaccharides (including, but not limited to, chitosan, chitin, alginic acid, starch, cellulose, konjac gums, etc.).^167^ On the other hand, synthetic biopolymers are obtained from natural polymers or chemically produced from synthetic monomers. In addition, they have the characteristic of natural manipulation without generating toxic waste for the environment. They are divided into two: nonbiodegradable biopolymers (e.g., PA, PVC, PP, PE, PMMA, polycarbonate, PU) and degradable biopolymers (e.g., PGA, PLA, PCL, PHB, PPF, PDS).^168^

From a functional perspective, biopolymers can be categorized as either biodegradable or nonbiodegradable, a classification that is determined by their response to degradation under physiological conditions. Biodegradable biopolymers naturally decompose without producing harmful substances; therefore, they are a natural alternative to petroleum-based polymers, which release toxic byproducts into the environment during their degradation.^169^ These classifications are fundamental to understanding how different materials behave in biological environments and selecting appropriate candidates for biomedical applications.

Chemically, they are composed of approximately ten monosaccharides linked by glycosidic bonds and are referred to as biomacromolecules. Typically, the monomeric units are composed of nucleic acids from nucleotides, proteins from amino acids, or saccharides derived from sugars.^170^ The performance of biopolymers is strongly governed by their molecular structure and physicochemical properties, including how these polymers organize into hydrated networks that resemble the ECM.^171,172^ From a molecular perspective, biopolymers are constituted of repeating units linked by covalent bonds, such as glycosidic linkages in polysaccharides and peptide bonds in proteins, forming macromolecular chains that can assemble into hydrogel networks.^171–173^ The architecture of these molecules may be linear or branched, and this influences properties such as solubility, chain entanglement, and network formation, which in turn affect mechanical behavior and pore architecture in the final hydrogel.^171,173,174^

The key physicochemical parameters that influence these interactions include hydrophilicity/hydrophobicity, surface charge, molecular weight, and polydispersity; these parameters affect interactions with water, ions, and biomolecules and thereby control swelling, diffusion, and mechanical properties.^173,175,176^ A salient characteristic of numerous biopolymers is their capacity to undergo gelation, thereby forming three-dimensional hydrated networks that are able to mimic the ECM; this process may occur through physical or chemical interactions and is influenced by environmental conditions such as pH, temperature, and ionic strength.^176,177^

Additional properties, including swelling behavior, permeability, and chemical stability, further determine the behavior of biopolymers in physiological environments and can be tuned via cross-linking density, composition, or hybrid designs.^171,173^ Collectively, these structural and chemical properties define the intrinsic behavior of biopolymers and their suitability for further functionalization, for instance, by introducing bioactive moieties or dynamic cross-links to achieve ECM-mimetic, stimuli-responsive, or injectable hydrogel systems.^173,175,176^

The overcoming of the inherent limitations of natural biopolymers, such as their low mechanical strength and poor printability, depends on the rational integration of chemical and physical modifications. A variety of strategies have been employed to modify rheological properties and gelation kinetics with the objective of enhancing structural stability and performance during the printing process. These strategies encompass methacrylation, oxidation, and photoinduced cross-linking.^178–180^

The formation of stable, printable networks is significantly influenced by physical approaches, including the control of pH, temperature, and ionic interactions.^179,181^ Recent studies demonstrate the efficacy of hybrid systems, combining biopolymers with inorganic phases or synthetic polymers, in achieving adequate mechanical performance without compromising biocompatibility.^178,179,182^

Furthermore, supramolecular modulation through the incorporation of architectures such as interpenetrating networks (IPNs) and double-network hydrogels has enabled significant enhancement of the mechanical strength and dimensional stability of printed constructs.^183^ The latest trends in the field focus on targeted functionalization strategies through the conjugation of bioactive peptides, thereby transforming biopolymers from passive materials into bioactive and smart platforms.^184–186^

Recent advances in biomaterial design have highlighted the strategic incorporation of bioactive motifs, such as RGD sequences (arginine–glycine–aspartic acid) and bone morphogenetic protein (BMP) mimetics, as powerful tools for the functional modification of polymers in tissue engineering. This enables precise control over cell adhesion, spreading, and mechanotransduction.^96^ The RGD motif constitutes a minimal recognition sequence derived from extracellular matrix proteins, which specifically binds to integrin receptors located on the cell membrane. When covalently grafted or physically immobilized onto polymeric backbones, it has been demonstrated that it can transform otherwise bioinert materials into biointeractive substrates, activating intracellular signaling pathways, including those involved in osteogenic differentiation.^92^

Concurrently, when BMP mimetics are conjugated to polymeric matrices, they have been observed to activate osteoinductive signaling pathways in a localized manner, thereby promoting the differentiation of mesenchymal stem cells into osteoblasts.^83,95^ These moieties are increasingly used to overcome the limitations associated with recombinant growth factors, including high cost, instability, and potential side effects. The combination of RGD-mediated adhesion cues with BMP-derived biochemical signals creates a synergistic microenvironment, where cell attachment and differentiation are spatially and temporally coordinated.^76^

## 5 Biopolymeric Bioinks

Biopolymers currently constitute the basis for many bone bioprinting systems. However, their practical application requires the development of bioinks with an appropriate biological performance and printability. In this context, biopolymers act as the base materials, whereas bioinks are processable formulations specifically designed for the controlled fabrication of biological constructs.^187^

Advances in bioink technology have enabled the fabrication of functional tissues and organ-like structures. Early studies introduced the terms “bioink” and “biopaper” in the context of organ printing research.^188^ Initially, the main approach involved printing a biopaper (hydrogel) followed by the deposition of living cells or tissue spheroids as bioinks. Subsequently, the concept evolved, and bioinks came to be recognized as structurally and functionally sophisticated materials capable of actively supporting tissue formation.^189^

Bioinks typically consist of one or more biomaterials, often hydrogels based on natural or synthetic polymers, and may also include ceramic phases, living cells, and/or bioactive components depending on the intended application.^190^ When cells are incorporated into bioinks, they become central components and must remain compatible with the automated biofabrication process.^191^ In bone regeneration applications, for example, biopolymeric bioinks may additionally incorporate stem cells, osteogenic factors, nanoparticles, ion-releasing phases, extracellular matrix components, or therapeutic molecules designed to enhance tissue repair. However, not all formulations used in biofabrication can be classified as bioinks. Cell-free systems containing biologically active molecules, thermoplastics loaded with active pharmaceutical ingredients, inorganic powders, or ion-releasing pastes are more appropriately described as biomaterial inks or bioprinting inks.^192^

In addition to being biocompatible, bioinks must exhibit desirable rheological behavior, good printability, shape retention after deposition, controllable cross-linking, structural stability, and the ability to support cellular activity during and after printing.^193^ Integrating all of these characteristics into a single formulation, however, remains challenging, as several of these requirements may conflict with one another. For instance, increasing viscosity may improve printing fidelity while reducing cell viability or hindering extrusion. Conversely, softer hydrogels may favor cell spreading but compromise the structural integrity of the printed construct.^74^ Therefore, the successful development of biopolymeric bioinks depends on balancing the processability with biological performance. Accordingly, these systems should be viewed as multifunctional, application-specific platforms designed to meet the complex demands of bone biofabrication.^193,194^

### 5.1 Physicochemical, Rheological, and Processing Parameters Governing Bioink Performance

Beyond the biological requirements for tissue regeneration, bioink performance is strongly influenced by physicochemical, rheological, and processing parameters, which should be evaluated in advance to understand the final behavior of 3D-printed biomaterials.^193,195^ These factors directly affect the printability, shape fidelity, structural stability, and cell survival during and after fabrication. Therefore, the selection of appropriate raw materials, high-quality cells, and the most suitable 3D printing technique is essential, since the fabrication method directly impacts structural resolution, cell viability, and the accuracy of the final construct.^196^

In this context, hydrogels are the most widely used matrices for bioinks because they provide a hydrated three-dimensional environment that supports cell encapsulation and facilitates the transport of oxygen, nutrients, and metabolic waste. They also help preserve an appropriate physiological microenvironment, contributing to osmotic balance and sustaining cellular activity after printing.^197^ In parallel, cell selection should consider origin, phenotype, and density, since incorporated cells must remain viable, functional, and capable of proliferation throughout the bioprinting process.^198^

Among the material-related properties, rheological behavior is particularly critical. Viscosity must be sufficiently high to maintain filament formation and structural fidelity after deposition, yet low enough to enable smooth extrusion and minimize shear-induced cellular damage.^193,196^ Accordingly, pseudoplastic (shear-thinning) behavior is highly desirable as viscosity decreases during printing and partially recovers afterward. Similarly, rapid viscoelastic recovery and an appropriate yield stress are advantageous for multilayer deposition and geometric stability.^199,200^

Polymer concentration and molecular weight strongly influence the rheological and mechanical properties of the bioinks. Increasing polymer content generally improves printability, shape fidelity, and mechanical strength; however, excessive concentrations may impair pore interconnectivity, nutrient diffusion, and cell spreading. Conversely, softer hydrogels may better support cellular activity and tissue-specific functions, but they often lack sufficient structural integrity to maintain complex printed architectures.^201,202^

Cross-linking is another key factor, as it stabilizes the printed structure after deposition. Physical cross-linking may occur through interactions such as electrostatic forces, hydrogen bonding, stereocomplex formation, hydrophobic associations, chain entanglements, and reversible crystallization.^203^ The efficiency of physical cross-linking depends on external stimuli such as temperature, pH, and ionic conditions. Divalent ions, including Ca^2+^, Ba^2+^, and Mg^2+^, are frequently used as physical cross-linking agents for alginate-based systems.

In contrast, chemical cross-linking involves the formation of permanent covalent bonds between polymer chains, resulting in stronger and more stable networks than those formed by physical cross-linking. Common chemical cross-linking systems include glutaraldehyde and carbodiimide-based approaches, although their applicability depends on the required biocompatibility and cytotoxicity profile.^105^

Beyond rheological and cross-linking properties, scaffold microarchitecture also plays a decisive role in bioink performance, particularly in bone regeneration. Parameters such as pore size, porosity, surface charge, and topography strongly influence cell adhesion, migration, nutrient diffusion, vascular infiltration, and mineralized tissue formation.^204,205^

Interconnected porous networks with appropriate pore dimensions facilitate osteoblast penetration and new bone ingrowth, while suitable porosity improves mass transport throughout the construct.^206^ In addition, surface topography can modulate cell orientation and differentiation, whereas surface charge affects protein adsorption, ionic interactions, and the initial cell adhesion. Therefore, controlling these structural and surface characteristics is essential for the successful development of bone-targeted bioinks.^207^

Overall, no single formulation can simultaneously optimize all of the properties required for an ideal bioink. Instead, successful bioink development relies on balancing processability, structural fidelity, biological performance, and application-specific functionality according to the requirements of each printing technique and regenerative objective.^208,209^ Figure 4 presents a schematic overview of the key properties of hydrogels and bioinks, with emphasis on porosity, pore size, surface charge, and topography.

**Figure 4:**
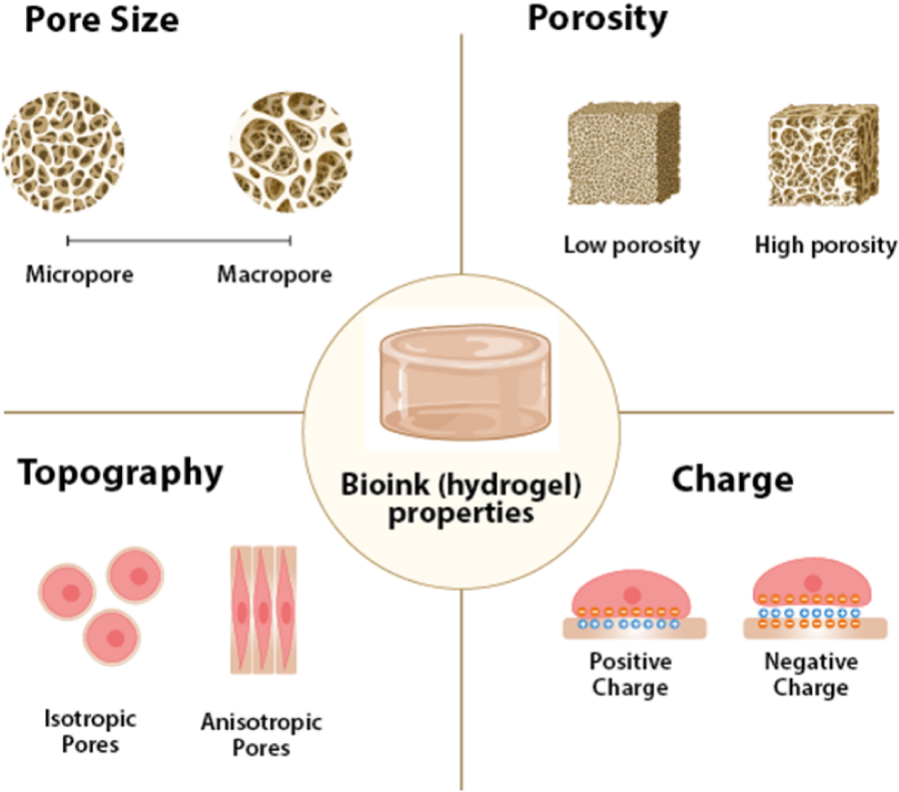
Schematic representation of the main physicochemical parameters of hydrogels and bioinks—pore size (micro/macropores), porosity, surface topography (isotropic/anisotropic), surface charge (positive/negative)—and their influence on cell–material interactions, mass transport, and overall functionality in tissue engineering.

## 6 Recent Advances in Biopolymer-Based Bioinks for Bone Regeneration

In recent years, bioinks have evolved from simple cell-laden hydrogels to multifunctional platforms capable of supporting structural integrity, promoting biological signaling, and enabling tissue regeneration, including biomaterial-assisted gene therapy and hydrogel-based systems for stem cell delivery. These approaches provide improved control over therapeutic targeting, gene expression, and cell release.^210,211^

This progress has been driven, for example, by the incorporation of composite materials, bioactive agents, and advanced biofabrication approaches.^199^ Additionally, advances toward microstructured and microenvironment-responsive systems have demonstrated an enhanced therapeutic efficacy. The integration of multiple bioactive agents, combined with biomimicry, targeted delivery, and controlled release, improves overall effectiveness and regenerative potential, driving the development of next-generation bioinks and biomaterials.^212–214^

Additionally, the biopolymers used in bioinks are capable of mimicking the ECM, are biocompatible, and possess tunable physicochemical properties—features that are highly desirable for biomaterials and tissue engineering applications. Despite these advances, several challenges, such as low mechanical stability, suboptimal printability, poor control over degradation kinetics, and difficulties in integrating vascularization and immune responses, are observed during the development of these materials and consequently hinder their clinical applications.^193,215^ Therefore, in order to improve the performance and functionality of biopolymer-based bioinks, new strategies have been explored, such as chemical modifications at the material level, the design of complex biomimetic architectures, and the development of bioactive systems.^216^

In this context, recent studies (2015–2025) can be categorized based on their main design strategies, including Mechanical and Rheological Enhancement Strategies, Structural and Multimaterial Approaches, Nanomaterial-Enabled Bioinks, Multifunctional and Therapeutic Bioinks, Biomimetic and Vascularized Constructs, and Smart and Immunomodulatory Bioinks for Microenvironment Regulation. The following sections critically discuss these approaches, highlighting their advantages, limitations, and future perspectives in the field of bone regeneration.

### 6.1 Mechanical and Rheological Enhancement Strategies

Although alginate-based bioinks are widely used because of their good biocompatibility, mild gelation conditions, and ease of processing, they still present important limitations for bone tissue engineering applications, particularly in situations requiring greater mechanical support. In general, their low mechanical strength, limited structural stability, and modest intrinsic bioactivity may compromise long-term functional performance and effectiveness.^217^ Thus, to improve the mechanical and rheological performance, several studies have explored the incorporation of reinforcing phases and complementary polymers.

Raja et al.^218^ developed a three-dimensional core–shell scaffold based on alginate, incorporating calcium-deficient hydroxyapatite (CDHA) and MC3T3-E1 preosteoblastic cells. The presence of the ceramic phase, combined with a hybrid dual-extrusion printing strategy, contributed to improved mechanical strength and osteoconductivity of the material. The uniaxial test of the core/shell scaffolds revealed a unique stress–strain curve when compared to the alginate scaffold. Furthermore, the core/outer layer scaffold also showed less detail deformation (32% by volume) when compared to pure alginate scaffolds (50% by volume), as it managed to counterbalance the low mechanical properties of the hydrogel and the fragility of precision while maintaining its 3D morphology.

Similarly, Bendtsen et al.^219^ combined alginate with hydroxyapatite (HA) and poly­(vinyl alcohol) (PVA), demonstrating that synthetic polymers can be used to modulate viscosity and improve printability while maintaining the biological activity. In this system, alginate was used as the hydrogel matrix base with the ability to encapsulate cells, PVA enhanced the structural stability and homogeneity of the bioink, and hydroxyapatite provided osteoconductive properties, promoting cell adhesion and bone regenerative potential. The developed system and the 3D bioprinting technique enabled the fabrication of structures with a uniform cell distribution and potential for bone regeneration.

In contrast, Tian et al.^220^ used alginate, gelatin, nanohydroxyapatite (n-HA), and human periodontal ligament stem cells (hPDLSCs) to obtain a bioink with cell adhesion capacity and osteogenic differentiation potential. In this case, the use of hydrophilic polymeric matrixes promoted high cell viability and nutrient diffusion. On the other hand, inorganic or bioactive phases increase stiffness and may induce osteogenic responses through specific physicochemical stimuli, with the incorporation of n-HA improving the compressive properties of the composite biological structures. In this study, the shear stress of the S3G7H5 composite hydrogel samples was higher than that of the S3 hydrogels at the same shear rate. At 60% deformation, the compressive stress of the S3 bioscaffold was lower than that of the S3G7H5 bioscaffold, showing a significant difference in mechanical properties between the two bioscaffolds (*P* < 0.01). Furthermore, the results showed that the SA/Gel/n-HA composite hydrogel exhibited good rheological properties, making it suitable for printing. The high swelling rate of the scaffold demonstrated that it possessed sufficient porosity and that the addition of n-HA improved the compressive properties of the composite bioscaffold. The cellular bioscaffold showed good biocompatibility and was beneficial to the osteogenic differentiation of hPDLSCs.

Özenler et al.^221^ developed a bioink based on fish scale (FS) and ADA-GEL with preosteoblast cells, showing uniform particle distribution and improved mechanical properties, including increased compressive strength and rigidity, while maintaining bioactivity and cytocompatibility. The printability of ADA-GEL/FS was evaluated in terms of Pr factor, U factor, pore size, and wire diameter. All groups of 3D-printed hydrogels exhibited a square pore shape, and Pr values were approximately one, indicating an ideal printing feature. Furthermore, the incorporation of FS particles did not alter the Pr and U factors, suggesting that the addition of FS did not affect printability, likely due to the elastic collagen fiber content of the particles. Viscosity increased with increasing amounts of FS in the ADA-GEL precursors, with a statistically significant difference, for example, when comparing groups with 3% FS (∼95 Pa·s), 5% FS (∼165 Pa·s), and 10% FS (∼232 Pa·s). All prepared materials exhibited shear-thinning behavior, indicating that all materials have extrusion printing capability. The thixotropic behavior of the groups containing ADA-GEL and 1% FS was similar, but groups with higher FS content showed increased recovery behavior. Furthermore, the incorporation of FS particles into the ADA-GEL matrix significantly increased the Young’s modulus of ADA-GEL, with the highest modulus recorded as 96 ± 8 kPa in ADA-GEL/10% hydrogels.

In this context, the combination of alginate matrices with calcium phosphate-based particles can improve structural fidelity, reduce postprinting deformation, and promote cell differentiation. Likewise, ceramic additives such as CDHA, HA, and nHA are among the most common strategies, as they mimic the mineral phase of native bone and improve compressive properties while providing osteoconductive cues. In parallel, secondary polymeric components such as PVA and gelatin have been used to optimize the rheological behavior, as these additives can improve viscosity control, extrusion stability, filament homogeneity, and cell-interactive properties. For example, gelatin contributes biological motifs favorable to cell adhesion, whereas synthetic polymers such as PVA primarily improve processing performance.^218–221^

Despite these advances, a relationship can be observed among mechanical reinforcement, printability, and biological performance. Although ceramic and mineral phases effectively increase stiffness and osteoconductivity, they may affect homogeneity and extrusion behavior.^222^ Polymeric and natural additives improve rheological properties and biocompatibility but generally provide limited mechanical support for load-bearing applications.^223^ Therefore, although composite strategies have improved the performance of alginate-based bioinks, achieving an optimal balance between structural integrity and biological functionality remains a critical challenge for their clinical applications.

### 6.2 Structural and Multimaterial Approaches

In addition to compositional modifications, structural design strategies have emerged as effective routes to overcome the intrinsic mechanical limitations of biopolymer-based bioinks. Instead of relying exclusively on changes in material formulation, these approaches use spatial organization, compartmentalization, and multimaterial integration to generate structures with improved mechanical stability while preserving biological functionality.^193,194^

A common concept is the separation of structural and biological functions within the same scaffold. Song et al.^224^ proposed a unit assembly model in which a polycaprolactone (PCL) framework provided external mechanical support, while an internal phase composed of alginate, tricalcium phosphate, and cells supplied the bioactive microenvironment. This strategy demonstrates how polymers with greater mechanical strength can compensate for the fragility of cell-laden hydrogels without compromising cell viability. Similarly, Raja et al.^225^ developed a core–shell construct combining a calcium phosphate ceramic core with an alginate hydrogel shell, enabling simultaneous mechanical reinforcement, cell encapsulation, and controlled release of therapeutic agents.

In another example, Janmohammadi et al.^226^ reported a multicompartment scaffold based on PCL and gum tragacanth, where the rigid polymer acted as a load-bearing framework, while the natural polymer phase promoted cell adhesion and osteogenic differentiation. Collectively, these studies demonstrate that architectural engineering may be as important as material composition in bioink design. By spatially distributing functions, multimaterial systems can achieve performance levels that are difficult to obtain with single-phase formulations.

However, these advantages come at the cost of a greater manufacturing complexity. Interfacial delamination, mismatched degradation kinetics, limited reproducibility, and increased process time remain significant barriers for scale-up and clinical translation.^227,228^ Therefore, future progress in this area will depend not only on smarter scaffold architectures but also on robust fabrication workflows capable of producing reproducible and clinically relevant constructs.

### 6.3 Nanomaterial-Enabled Bioinks

The incorporation of nanomaterials into biopolymer-based bioinks has emerged as a strategy to simultaneously improve mechanical performance, rheological behavior, and biological functionality.^229^ Beyond passive reinforcement, nanomaterials can actively modulate the cellular microenvironment, enable the controlled release of bioactive factors, and confer multifunctionality to printed constructs.^230^

Among the most widely explored systems, nanosilicates have shown particular relevance because of their dual rheological and osteogenic effects. Liu et al. developed an alginate/gelatin bioink containing nanosilicates, in which the nanoparticles improved shear-thinning behavior, print fidelity, and compressive modulus while simultaneously promoting osteogenic differentiation without exogenous growth factors. The slow degradation and facile ionic cross-linking of alginate ensured structural retention in vivo. These findings suggest that nanosilicates may act not only as reinforcing agents but also as bioactive cues. Nanofillers, such as laponite, have been investigated as carriers for therapeutic molecules.

Cidonio et al.^231^ incorporated laponite into gellan gum bioinks, resulting in improved rheological stability and sustained release of vascular endothelial growth factor (VEGF), which enhanced the angiogenic responses in vivo. Therefore, this reinforces the importance of nanofillers, particularly in systems that require the controlled release of growth factors. In contrast, carbon-based nanomaterials are often prioritized for mechanical reinforcement and interfacial interactions.

Zhu et al.^232^ combined graphene oxide with gellan gum, curcumin (Cur), and a human osteosarcoma cell line (MG-63). The 3D-printed scaffolds exhibited an interconnected porous structure, suitable mechanical properties, high print fidelity, controlled Cur release, and promotion of cell adhesion and proliferation. However, although graphene-derived materials offer significant functional advantages, they often raise greater concerns regarding their long-term biosafety and dose-dependent cytotoxicity.

In this context, these different nanomaterials offer distinct advantages: nanosilicates are attractive for enhancing osteogenesis and rheological properties, laponite is highly effective for the controlled release of bioactive factors, and graphene oxide provides strong mechanical reinforcement and multifunctional surface interactions.^232^

Despite these advances, several important challenges still need to be addressed, including nanoparticle aggregation, batch-to-batch variability, uncertainties regarding long-term biodistribution, and potential cytotoxic effects.^233^ Moreover, the nanoparticle concentrations required to maximize stiffness may simultaneously impair cell viability or extrusion behavior.^234^ Therefore, the advancement of nanomaterial-based bioinks will depend on the ability to carefully balance functional performance, biological safety, and reproducibility in the manufacturing process.^229^

### 6.4 Multifunctional and Therapeutic Bioinks

In contrast to conventional bioinks, which are primarily designed for structural support, multifunctional and therapeutic bioinks actively participate in tissue regeneration by modulating the local biological environment.^235^ These advanced systems incorporate bioactive molecules or therapeutic agents capable of promoting osteogenesis, angiogenesis, antimicrobial protection, and immunoregulation, thereby expanding the regenerative role of the printed constructs. An important strategy involves the incorporation of anti-inflammatory proteins or signaling modulators.^236,237^

Wang et al.^238^ developed an alginate/nanohydroxyapatite scaffold loaded with Atsttrin, a progranulin-derived protein fragment capable of antagonizing TNF-α signaling. After 3D printing, the system exhibited sustained release for up to 5 days, low cytotoxicity, good cell adhesion, and enhanced osteogenic activity, demonstrating that the control of inflammatory pathways can directly improve bone repair outcomes. A second major approach focuses on angiogenic stimulation.

Ahlfeld et al.^239^ developed a biphasic construct combining calcium phosphate cement with an alginate/gellan gum hydrogel loaded with VEGF. This strategy combined the mechanical and osteoconductive advantages of the ceramic phase with the localized release of provascular signals, promoting improved vascularization and bone formation. The CPC demonstrated excellent osteoconductivity, while the local release of VEGF by the AlgGG hydrogel promoted vascularization, as indicated by a greater number of blood vessels compared to the CPC and CPC/AlgGG groups. In in vitro culture experiments with rat MSCs, cells were able to adhere, spread, and proliferate not only on the surface of CPC but also on the surfaces of AlgGG fibers. In this study, the hydrogel component did not affect regenerative processes in the defect region; however, fixation of growing bone, as well as osteoclastic activity, was observed only in CPC filaments, but not in filaments made of AlgGG. The authors attribute this to the fact that alginate cannot be enzymatically cleaved by mammalian cells, with its degradation resulting from the disintegration of the cross-linked hydrogel network by the release of calcium ions and subsequent hydration and swelling. However, for gellan gum, it has been reported that in vivo degradation is a slow process of hydrolytic degradation and ion exchange; however, due to its low concentration in the AlgGG mixture, no notable change was detected in the AlgGG filaments. Therefore, a critical analysis is important because, although alginate-based hydrogels offer high biocompatibility and bioprinting capabilities along with bioactive growth factors and cells, limited degradation must be evaluated for the application of alginate-based biomaterial inks in implants for bone regeneration.

Small therapeutic molecules have also emerged as attractive alternatives to recombinant proteins. Li et al.^101^ incorporated deferoxamine (DFO), a hypoxia-mimicking agent, into printed constructs to activate the HIF-1α pathway. The sustained release of DFO by the scaffold will positively affect the synergistic effects of vascularization and osteogenic differentiation during bone repair; all groups exhibited some cell migration, while the Eth-DFO@GelMA/GGMA group showed the most pronounced effect on cell migration and near-healed wounds. The VEGF concentrations detected in the GelMA, GelMA/GGMA, and Eth-DFO@GelMA/GGMA groups were 172.21 ± 5.26, 189.84 ± 2.89, and 233.79 ± 10.53 ng/L, respectively, being significantly higher than in the control group (87.47 ± 1.05 ng/L), while the Eth-DFO@GelMA/GGMA group was more effective in promoting VEGF expression. In vivo experiments in a rat model of cranial defects demonstrated that the composite scaffold can promote angiogenesis and bone regeneration by activating the hypoxia-inducible factor 1α (HIF1-α) signaling pathway. When the HIF1-α signaling pathway is activated, it can initiate the expression of its downstream genes, such as eNOS, VEGF, and SDF1, which are key regulators of blood vessel growth. Therefore, these results suggest that the Eth-DFO@GelMA/GGMA scaffold can effectively release DFO to activate the HIF1-α signaling pathway, which, in turn, regulates the expression of genes related to angiogenesis.

More recently, extracellular vesicle-based systems have attracted considerable attention. Kang et al.^240^ developed a hybrid bioink composed of decellularized extracellular matrix (dECM), gelatin, quaternized chitosan (QCS), and nanohydroxyapatite (nHAp), functionalized with mesenchymal stem cell-derived exosomes. The system was found to promote cell proliferation, vascularization, and bone regeneration both in vitro and in vivo. In addition, it exhibited structural, antibacterial, and bioactive properties, in which QCS provided antimicrobial activity, nHAp improved mechanical strength and osteoconductivity, while dECM offered a biomimetic microenvironment. However, exosome-based therapies still face challenges related to standardization, large-scale production, storage stability, and regulatory approvals.

Overall, these studies show that no therapeutic strategy is superior in all contexts. Protein-based systems provide strong biological signaling but present limitations related to cost and stability.^241^ Small molecules are more practical and scalable but require precise control of their release. Exosome-based platforms expand regenerative potential, although they remain technologically complex.^242^ Thus, the future of therapeutic bioinks is likely to involve hybrid systems that combine structural support, biological signaling, and translational feasibility.^193^

### 6.5 Biomimetic and Vascularized Constructs

One of the major advances in biopolymer-based bone bioinks is the attempt to reproduce two essential features of native bone: its hierarchical architecture and its tightly coupled angiogenic–osteogenic microenvironment.^243^ Thus, the development of biomimetic and vascularized constructs is important, since successful bone regeneration depends on osteogenesis and the rapid establishment of a functional vascular network.^244^

A representative example of structural biomimicry was reported by Ghahri et al.,^245^ who developed an osteon-like construct using a quadruple coaxial extrusion system. For this purpose, a composite hydrogel based on GelMA, alginate, and gelatin was used, incorporating HA, whitlockite, PLGA nanoparticles loaded with VEGF, as well as PEG as a sacrificial agent and CaCl_2_ for cross-linking. The system recreated key features of cortical bone by integrating a central Haversian-like channel surrounded by concentric cell-laden layers with vasculogenic and osteogenic compartments. This study illustrates how spatial organization can be used to emulate the native compartmentalization of the bone tissue.

In contrast to structural biomimicry, Shen et al.^246^ emphasized functional vascularization through in situ endothelialization. For this purpose, they adopted a dual-bioink strategy involving the coprinting of a GelMA matrix loaded with BMSCs and a thermosensitive bioink loaded with endothelial cells. This method helps improve the uneven distribution of endothelial cells within scaffolds by enabling spatially controlled cell deposition during fabrication. Thus, this approach promoted osteogenic differentiation through angiogenesis–osteogenesis coupling and improved bone regeneration in critical-sized cranial defects. Importantly, the comparison between these studies suggests that successful biomimicry does not necessarily depend on exact structural replication, but rather on reproducing essential biological functions. In this context, function-oriented vascular integration may sometimes be more clinically relevant than strict anatomical fidelity.

More translationally oriented approaches have sought to reduce the dependence on exogenous cells and growth factors. Yang et al.^247^ developed a cell-free oxygen-generating hydrogel scaffold capable of promoting vascularized bone repair by alleviating local hypoxia and stimulating endogenous angiogenesis. Carboxymethyl cellulose and polyacrylamide cross-linked with CaCl_2_ were used, together with CaO_2_ nanoparticles encapsulated within ZIF-8 structures. The synergistic effect of oxygen supply and a hierarchically porous architecture facilitated both vascular growth and bone formation even in the absence of cells or growth factors. This strategy offers lower regulatory complexity and greater practicality, although it may provide a lower biological precision in severely compromised tissues.

Similarly, Cao et al.^248^ proposed a PRP-based vascularized bioink combining GelMA/AlgMA, laponite nanoparticles, and PCL reinforcement. In this system, platelet-rich plasma served as an autologous source of growth factors, while laponite modulated the release kinetics, and PCL improved mechanical stability. The construct promoted endothelialization, osteogenesis, and macrophage polarization toward a regenerative phenotype. However, variability among donors and the inconsistent composition of PRP remain challenges for reproducibility.

In general, these studies show that isolated strategies have difficulty fully reproducing the complexity of native bone. Highly biomimetic constructs tend to better replicate tissue architecture but face challenges related to scalability and increased manufacturing complexity.^249^ In contrast, simpler or acellular systems are more practical, although they typically offer less biological control.^250^ Thus, future bioinks will likely depend on hybrid approaches capable of combining structural guidance, controlled signaling, and host-mediated vascularization mechanisms, thereby integrating functional performance with translational feasibility.^251^

### 6.6 Smart and Immunomodulatory Bioinks for Microenvironment Regulation

The development of smart bioinks containing immunomodulatory agents represents a significant advance, as these formulations can actively modulate the tissue microenvironment and direct the inflammatory response to promote regenerative processes.^150^

For this purpose, resources are employed that include bioactive factors, signaling molecules, and multifunctional nanocomposites.^252^ Grandjean et al.^253^ developed a bioactive bioink based on alginate and methylcellulose, loaded with Platelet-Rich Fibrin (PRF) concentrate (PRF/iPRF). The bioinert biopolymer (alginate) was functionalized with biological components but without active control over the behavior of immune cells. In this case, the immunomodulatory role was indirect, mediated by the release of proangiogenic growth factors (such as VEGF and TGF-β), which promote regeneration and blood vessel formation.

On the other hand, Dutta et al.^254^ developed a bioinstructive bioink capable of directly controlling the immune response based on alginate, gelatin, collagen, and dECM, combined with polydopamine nanoparticles and exosomes derived from M2 macrophages. In addition to providing structural support, this bioink actively modulates the immune microenvironment by promoting macrophage polarization toward the M2 phenotype and regulating inflammatory and angiogenic pathways. This latter approach offers more precise control of the regenerative microenvironment.

Another advance is smart bioinks, marking the shift from passive to actively modulatory systems.^150^ One example was the study conducted by Qiu et al.,^255^ a next-generation immunomodulatory nanocomposite scaffold enabling macrophage-mediated mitochondrial transfer to enhance osteogenesis, in which they developed a gelatin/alginate-based hydrogel modified with dopamine and silver-decorated iron oxide nanoparticles (GAD/Ag-pIO). Its main innovation was the integration of antioxidant activity, immunomodulation, and stimulation of mitochondrial transfer from macrophages to bone marrow mesenchymal stem cells (BMSCs), thereby enhancing the osteogenic differentiation and promoting bone repair.

In a more translational approach, Xingge Yu et al.^256^ used GelMA as the matrix biopolymer in combination with strontium-substituted Xonotlite nanowires (Sr-CSH) and encapsulated BMSCs. The ceramic component improved the rheological and mechanical properties of the bioink, while the release of bioactive ions promoted macrophage polarization toward the M2 phenotype and supported complete regeneration in critical-sized bone defects.

Complementarily, San-yang Yu et al.^257^ used GelMA functionalized with metal–organic framework nanoparticles (LUT@ZIF-8), enabling the sustained release of luteolin and Zn^2+^. According to the results, the system exhibited simultaneous antibacterial action, anti-inflammatory activity, and osteogenic stimulation, in addition to promoting the M1-to-M2 transition, thereby representing a more sophisticated multifunctional strategy for microenvironmental control. In light of this, the studies show that the effectiveness of next-generation bioinks depends on the integration of printable biopolymers, bioactive agents, and precise modulation of the macrophage response to enhance osteogenesis.

Compared with the immunomodulatory bioinks discussed earlier, Kim et al. (2024)^258^ shifted the focus from macrophage regulation to pharmacokinetic control and therapeutic compartmentalization by developing an advanced functional bioink designed for the spatially controlled delivery of osteoinductive factors. The system consisted of a bilayer 3D-printed scaffold that combined polycaprolactone (PCL) as the structural phase and physical barrier, a gelatin/alginate hydrogel as the bioink, and fibroin lipoplex nanoparticles functionalized with the osteotropic peptide DSS6 and loaded with BMP-2. This innovation aimed to integrate multilayer bioprinting, bone targeting, and a prolonged BMP-2 release into a single platform. Accordingly, this architecture was found to provide high protein stability, bone-specific retention, and sustained release of the growth factor. Through in vivo analysis, superior regeneration was observed in critical-sized calvarial defects. In addition, the PBN scaffold prolonged BMP-2 release by more than 8-fold, increased its local concentration at the defect site, and reduced diffusion into soft tissues, thereby minimizing ectopic ossification and peripheral inflammation.

Therefore, the future of bone biofabrication depends on the development of smart bioinks capable of dynamically regulating immunological, biochemical, and spatial signals to promote safer and more effective regeneration.^259^ Several research groups are currently focused on developing biopolymer-based bioinks for application in 3D bioprinting technology to produce materials that assist bone tissue regeneration. Thus, the materials, cells, main findings, and different printing parameters of the analyzed studies (2015–2025) are presented in Table 1, and a comparative analysis of the most commonly used biopolymers in bone bioprinting is shown in Table 2.

**Table 1:** Recent Advances in Biopolymer-Based Bioinks for Bone Regeneration Based on Biopolymers Discussed in the Present Study (2015–2023)

Biopolymer	Other materials	Cells	Bioactive factor	Type of 3D bioprinting	Biomaterial developed	3D printing parameters	Key findings	Ref
Alginate	α-TCP (CDHA-forming bioceramic); HPMC (rheology modifier); CaCl_2_ (cross-linker)	Mouse preosteoblast (MC3T3-E1)	None (bioactivity from CDHA phase)	Dual extrusion (core/shell bioprinting; PED-based system combining screw + pneumatic extrusion)	Hybrid core–shell scaffold (ceramic core + cell-laden hydrogel shell)	Core nozzle: 300–700 μm; Shell nozzle: 1000–1200 μm; screw speed: 400 rpm (core); pneumatic pressure: ∼110–117 kPa (shell); printing speed: 4.8–7.8 mm/s; alginate: 9 wt %; CaCl_2_ cross-linking: 0.75% (pre) + 2.5% (post, 30 min); PBS incubation: 6 h; full cementation: ∼72 h in medium	Core–shell structure improved mechanical strength and stability; high long-term cell viability; tunable mechanics; enhanced structural integrity	^218^
Alginate	PVA (viscosity modifier); HA (osteoconductive phase); Ca^2+^ salts (cross-linkers)	Mouse preosteoblasts (MC3T3-E1)	None (intrinsic osteoconductivity from HA)	Extrusion-based 3D bioprinting (pneumatic/modified extruder system)	Cell-laden composite hydrogel scaffold (alginate–PVA–HA)	Nozzle: 23G (∼430 μm); gelation time: ∼20 min preprinting; scaffold: 7 layers, Ø 1.5 cm; cross-linking: Ca^2+^ (CaSO_4_ + CaCl_2_ bath); optimal storage modulus: 600–1200 Pa	Improved printability and rheology; high cell viability; enhanced osteoconductivity; stable constructs	^219^
Alginate; Gelatin	nHA (osteoconductive phase)	Human periodontal ligament stem cells (hPDLSCs)	None (intrinsic osteoinductivity from nHA)	Extrusion-based 3D bioprinting (pneumatic bioplotter)	Cell-laden composite hydrogel scaffold (SA/Gel/nHA)	Nozzle: 400 μm; speed: 6 mm/s; pressure: 0.2 MPa; tube temp: 25 °C; platform temp: 4 °C; cross-linking: CaCl_2_ (2% w/v, ∼180 mM, 5 min); composition: SA 3%, Gel 7%, nHA 5%	Enhanced mechanical properties; reduced swelling; improved cell adhesion and osteogenesis	^220^
Alginate dialdehyde; Gelatin	Fish scale particles (collagen/HA-based bioactive filler)	Mouse preosteoblast (MC3T3-E1)	None (intrinsic bioactivity from fish scale particles)	Extrusion-based bioprinting (pneumatic)	Composite hydrogel (ADA-GEL/FS) with particulate reinforcement	Nozzle: 400 μm; speed: 10 mm/s; pressure: 100–190 kPa; temp: 30 °C; 10 layers; cross-linking: CaCl_2_ (ionic) + mTG (enzymatic)	Increased stiffness and bioactivity; high cell viability; enhanced osteogenic differentiation	^221^
Alginate	PCL (structural support); TCP (osteoconductive phase)	Human hepatocarcinoma cells (BEL-7402)	None (bioactivity from TCP phase)	Hybrid bioprinting: extrusion (PCL) + pneumatic extrusion/air-pressure dispensing (cell-laden bioink)	Hybrid scaffold with PCL supportive frame + TCP/alginate bioink filler (unit-assembly modular construct)	PCL: 90 °C, nozzle 0.33 mm, speed 5 mm/s, pressure 0.3 MPa; Bioink: nozzle 19G (∼0.9 mm), pressure 10–130 kPa; alginate 2–6% (w/v); cross-linking: 0.1 M CaCl_2_ (20 min); printing *T* = 37 °C	Improved mechanical properties via PCL support; high printability; good cell viability; modular fabrication	^224^
Alginate	α-TCP (CDHA-forming bioceramic); HPMC (rheology modifier); quercetin (osteogenic agent)	Mouse preosteoblast (MC3T3-E1)	Quercetin (osteogenic agent)	Extrusion-based bioprinting (concentric/coaxial nozzle system)	Core–shell hydrogel–ceramic microbeads (multifunctional system)	Inner nozzle: 300–500 μm; alginate: 1–2 wt %; extrusion speed: 2–4 mm/min; cross-linking: CaCl_2_ (140 mM); cementation in PBS (∼6 h); *T* = 37 °C	Core–shell microbeads enabled sustained drug release (∼120 days); high cell viability; enhanced osteogenesis	^225^
Tragacantha Gum	PCL (structural support); 45S5 bioactive glass (ion-releasing phase)	Mouse preosteoblast (MC3T3-E1)	None (ion release from bioactive glass)	Extrusion-based 3D printing (PCL framework) + postfabrication filling (freeze-dried TG-BG)	Multicompartment host–guest scaffold (polymer framework + bioactive matrix)	PCL printing: 90 °C; strut ∼400 μm; pore size ∼700 μm; 0–60–120° laydown pattern; porosity ∼54% (host); final porosity >60%	Enhanced mechanical strength and bioactivity; promoted osteogenesis and mineralization; biomimetic structure	^226^
Alginate; Gelatin	Nanosilicate (nSi, rheology modifier and osteoinductive agent)	Rat bone marrow mesenchymal stem cells (rBMSCs)	None (bioactivity from nanosilicate ions)	Extrusion-based bioprinting	Nanocomposite ECM-mimetic bioink	Nozzle: 22G; speed: 50 mm/s; layer height: 300 μm; flow: 12.72 mL/h; cross-linking: CaCl_2_ (10 min)	Improved printability and mechanical strength; induced osteogenesis without supplements; enhanced bone regeneration in vivo	
Gellan Gum	Laponite (nanoclay, rheology modifier); CaCl_2_ (cross-linker)	Mouse myoblast cells (C2C12)	VEGF (angiogenic factor)	Extrusion-based free-form bioprinting (printing in support bath)	Nanocomposite bioink (GG + nanoclay) with growth factor delivery	Nozzle ∼250 μm; speed: 2.5 mm/s; layer height: 300 μm; cross-linking: CaCl_2_ (10 min); printing in agarose fluid ge	Improved printability and shape fidelity; sustained VEGF release; enhanced angiogenesis and osteogenesis	^231^
Gellan Gum	Graphene oxide (mechanical reinforcement)	Mouse osteoblast cell (MC3T3); Human osteosarcoma cells (MG-63)	Curcumin (therapeutic agent)	Extrusion-based 3D printing	Drug-loaded multifunctional hydrogel scaffold (tumor therapy + bone regeneration)	Nozzle: 22G (∼0.41 mm); temp: 37 °C; platform: 25 °C; speed: 10 mm/s; postcross-linking: CaCl_2_ (10 min)	Improved mechanical properties; sustained drug release; promoted osteoblast proliferation and inhibited tumor cells	^232^
Alginate; Gelatin	nHA (osteoconductive phase)	Murine mesenchymal stem cells (C3H cells)	Atsttrin (anti-inflammatory agent)	Pneumatic extrusion bioprinting	Composite hydrogel–ceramic scaffold (anti-inflammatory)	Nozzle: 100 μm; strand: ∼100 μm; pore spacing: ∼400 μm; multilayer scaffold; CaCl_2_ + EDC cross-linking	Sustained anti-inflammatory factor release; reduced TNF-α; enhanced osteogenesis and bone formation	^238^
Alginate; Gellan Gum	CPC (osteoconductive phase)	Rat mesenchymal stromal cells (MSC); human MSC; HUVEC	VEGF (angiogenic factor)	Extrusion-based multichannel plotting	Biphasic scaffold (ceramic + hydrogel reservoir)	Nozzle: ∼230–250 μm; layer-by-layer deposition; postprocessing: CaCl_2_ (10 min) + CPC setting (3 days)	Sustained VEGF release; enhanced angiogenesis; limited effect on bone formation	^239^
Gelatin methacrylate; Gellan Gum methacrylate	Ca^2+^ (ionic cross-linker); LAP (photoinitiator); ethosomes (drug carriers)	Human umbilical vein endothelial cells (HUVEC); Rat bone marrow mesenchymal stem cells (rBMSC)	Deferoxamine (DFO, pro-angiogenic agent)	Extrusion-based bioprinting (pneumatic)	Double-cross-linked hybrid hydrogel (photo + ionic) with drug delivery system	Pressure: ∼0.15 MPa; nozzle: 0.26 mm; temp: ∼20 °C; speed: ∼2 mm/s; layer height: 0.15 mm; filament spacing: 0.8 mm; cross-linking: 405 nm light + CaCl_2_ (0.3 M)	Sustained DFO release; enhanced angiogenesis and osteogenesis; improved bone regeneration in vivo	^101^
Decellularized ECM; Gelatin; Quaternized chitosan	nHA (osteoconductive phase); exosomes (bioactive vesicles); EDC/NHS (cross-linking system)	None (cell-free system; hADSC-derived exosomes)	MSC-derived exosomes (paracrine signaling factors)	Extrusion-based 3D bioprinting (pneumatic-driven)	Hybrid multifunctional nanocomposite scaffold (dECM/Gel/QCS/nHAp@Exo)	Nozzle temp: 20–25 °C; platform temp: 10 °C; pressure: 0.24–0.27 MPa; speed: 8–10 mm/s; needle: 0.31 mm; filling distance: 0.7 mm; cross-linking: EDC/NHS (6 h)	Enhanced cell adhesion and antibacterial activity; sustained exosome release; promoted angiogenesis and osteogenesis	^240^
Alginate; Gelatin methacrylate; Gelatin	WH/HAP nanoparticles (osteoconductive phase); PLGA (drug carrier)	Human umbilical vein endothelial cells (HUVECs); human mesenchymal stem cells (hMSCs)	VEGF (angiogenic factor)	Extrusion-based quadruple coaxial bioprinting (core–shell multilayer)	Osteon-like multilayer hollow scaffold (vasculogenic + osteogenic layers)	Speed (5 mm/s); Flow rate (internal to external: 0.2, 0.3, 0.1, and 0.4 mL/min); Layers with cubic dimensions of 20 × 20 × 4 mm and fiber spacing of 1.5 mm; 30s UV exposure for GelMA cross-linking	High cell viability; vascular channel formation; enhanced osteogenesis and mineralization; sustained VEGF release	^249^
Gelatin methacrylate	PLA–PEG–PLA (structural polymer); LAP (photoinitiator)	Rat bone marrow mesenchymal stem cells (rBMSCs); rat aortic endothelial cells (RAOECs)	Endogenous factors (VEGF, PDGF, HIF-1α)	Extrusion-based 3D bioprinting (dual-nozzle)	Hybrid hydrogel (cell-laden composite scaffold)	Layer height: 0.2 mm; extrusion width: 0.2 mm; speed: 600 mm/min; pressure: 0.05–0.09 MPa; nozzle temp: 22–25 °C; bed temp: 5 °C	Enhanced vascularization and osteogenesis; improved bone regeneration in vivo	^246^
Gelatin methacrylate; Methacrylated Alginate	PCL (structural support); Nanoclay (rheology modifier)	Rat bone marrow mesenchymal stem cells (rBMSCs); HUVECs; RAW264.7 macrophages	PRP-derived growth factors (VEGF, PDGF, TGF-β)	Extrusion-based 3D bioprinting (hydrogel + PCL hybrid printing)	Hybrid composite scaffold (natural-derived hydrogel + synthetic polymer + nanoclay)	Needle diameter: 250 μm; temperature: 12–20 °C (nozzle), platform ∼4 °C lower; speed: 10 mm/s; pressure: 0.16 MPa; UV curing: 405 nm; CaCl_2_ cross-linking; PCL nozzle: 300 μm at 60 °C	Sustained oxygen and ion release; enhanced angiogenesis and osteogenesis; complete bone regeneration without cells	^248^
Sodium carboxymethylcellulose	ZIF-8 (ion-releasing MOF); CaO_2_ (oxygen-releasing agent)	Mouse preosteoblasts (MC3T3-E1); human umbilical vein endothelial cells (HUVECs)	Oxygen (O_2_ release); Zn^2+^ ions	Extrusion-based 3D printing (UV-assisted)	Hybrid double-network hydrogel + nanoparticle composite (cell/factor-free scaffold)	Nozzle: 300 μm; speed: 50 mm/s; extrusion rate: 4 mm/s; UV curing: 405 nm; layer rotation: 90°; room temperature printing	Sustained growth factor release; improved angiogenesis and cell proliferation; high biocompatibility	^247^
Sodium alginate; Methylcellulose	i-PRF (autologous growth factor source)	SaOS-2; HUVECs; L929 fibroblasts	VEGF; PDGF; TGF-β (from PRF)	Extrusion-based bioprinting (pneumatic)	Hydrogel-based bioink (alginate/cellulose + autologous platelet concentrate)	Nozzle: 0.25 mm; speed: 10–12 mm/s; pressure: 70–80 kPa; cross-linking: 100 mM CaCl_2_ (10 min); infill: 20% honeycomb	Sustained growth factor release; enhanced angiogenesis; preserved bioactivity after printing	^253^
Alginate; Gelatin; Collagen; Decellularized ECM (skin-derived)	PDA nanoparticles (bioactive coating); exosomes (immunomodulatory factors)	RAW 264.7 macrophages; hDFs; keratinocytes (HaCaT); hBMSCs; endothelial cells	M2-derived exosomes (immunomodulatory factors)	Extrusion-based bioprinting (pneumatic, BIO-X) + support bath printing	Composite bioactive hydrogel (dual-bioink system: immunomodulatory + skin bioink)	Nozzle: 250 μm (25G); speed: ∼5.5 mm/s; infill: 20%; temperature: 37 °C; cross-link: CaCl_2_ (for alginate phase); support bath: Pluronic 10–15%	Enhanced angiogenesis and wound healing; reduced inflammation; improved tissue regeneration	^254^
Dopamine-modified Alginate; Gelatin; Collagen	Ag-pIO nanoparticles (antibacterial/ROS-scavenging agent)	BMSCs; bone marrow-derived macrophages (BMDMs)	None (ROS-scavenging and immunomodulatory effects)	Extrusion-based 3D printing	Immunomodulatory nanocomposite hydrogel scaffold	Nozzle/syringe temperature: 31 °C; platform temperature: 4 °C; pressure/speed optimized according to Gel/Alg-DA ratio: 170/15, 220/12, 280/12, and 360/15 kPa/mm s^–1^; final ratio Gel/Alg-DA = 70/30; 5 wt % Ag-pIOPNs; 1–10 layers; cross-linking with 1% CaCl_2_ for 2 min	Reduced inflammation and ROS; promoted M2 polarization; enhanced osteogenesis and bone healing	^255^
Gelatin methacryloyl	Sr-CSH (ion-releasing phase)	Rat bone marrow mesenchymal stem cells (rBMSCs); RAW 264.7 macrophages	Bioactive ions (Sr^2+^, Ca^2+^, Si^4+^)	Extrusion-based 3D bioprinting	Osteoimmunomodulatory nanocomposite hydrogel bioink	Bioink: 10% GelMA + 5% Sr-CSH; pregelation at 4 °C (10 min); printing temperature: ∼15–20 °C; pressure: 0.15–0.2 MPa; speed: 8 mm/s; needle: 22G; cross-linking: 405 nm blue light (60 s); cell density: 2 × 10^6^ cells/mL	Induced M2 macrophage polarization; enhanced osteogenesis and mineralization; improved bone regeneration	^256^
Gelatin methacryloyl	ZIF-8 (drug carrier)	Rat bone marrow mesenchymal stem cells (rBMSCs); RAW 264.7 macrophages	Luteolin (anti-inflammatory agent); Zn^2+^ (bioactive ion)	Extrusion-based 3D bioprinting	MOF-functionalized osteoimmunomodulatory hydrogel scaffold	Bioink: GelMA + LAP + LUT@ZIF-8; printing temperature: syringe 18–22 °C, platform 18 °C; extrusion speed: 2 mm/min; printing speed: 5 mm/min; nozzle: 23G; UV cross-linking: 405 nm (0.5 W/cm^2^); cell density: 1.5 × 10^6^ cells/mL	Sustained drug release; antibacterial activity; enhanced osteogenesis and bone regeneration	^257^
Gelatin; Alginate	PCL (structural support)	None (cell-free scaffold; in vitro osteogenic assays)	BMP2 (osteogenic growth factor); DSS6 (bone-targeting peptide)	Extrusion-based 3D bioprinting (bilayer scaffold fabrication)	Bilayer composite scaffold (PCL + bioink + nanoparticle-lipoplex system)	PCL printing: nozzle 100 μm; temp 83 °C; bed 24 °C; fill density 10–30%; bioink: Gel (20%) + Alg (8%), CaCl_2_ cross-linking; nanoparticle loading ∼10% (v/v)	Sustained BMP2 release (>30 days); targeted delivery; enhanced osteogenesis; improved bone regeneration	^258^

**Table 2:** Comparative Analysis of Commonly Used Biopolymers in Bone Bioprinting (2015–2025)

Biopolymer	Representative studies	Printability	Mechanical properties	Biological performance	Osteogenic potential	Main advantages	Main limitations
Alginate	^218,219,224,225^	High	Low	Low	Low	Excellent printability; rapid ionic cross-linking; easy processing; widely used	Poor cell adhesion; bioinert; weak mechanical strength; requires modification
Alginate + Gelatin	^220,238^	High	Moderate	High	Moderate	Improved cell adhesion (RGD motifs); better bioactivity; ECM-mimetic environment	Limited mechanical strength; fast degradation; often requires reinforcement (e.g., nHA)
Alginate derivatives (ADA, Alg-DA)	^221,255^	High	Moderate	High	High	Tunable cross-linking (Schiff base); improved cell interaction; multifunctionality (immunomodulation)	More complex synthesis; stability dependent on formulation
Gellan gum	^231,232^	High	Moderate	Moderate	Moderate	High shape fidelity; strong rheological properties; stable structures	Limited intrinsic bioactivity; requires additives (e.g., nanoclay, drugs)
GelMA (and derivatives)	^101,245,256^	Moderate	Low	Very high	High	Excellent cell interaction; supports angiogenesis; tunable via photocross-linking	Poor mechanical properties; requires reinforcement or hybrid systems
dECM-based bioinks	^240,254^	Low	Low	Very high	High	Highly biomimetic; promotes cell adhesion and signaling; strong biological relevance	Poor printability; variability; low mechanical stability
Chitosan/derivatives (QCS, CMC)	^240,247^	Low	Low	High	Moderate	Antibacterial; bioactive; supports osteogenesis and immunomodulation	Poor solubility; limited printability; requires blending
Tragacanth gum	^226^	Moderate	Moderate	High	High	Natural bioactivity; supports osteogenic differentiation; ECM-like behavior	Limited studies; requires combination with other materials
Multicomponent Hybrid systems (e.g., Alg/GelMA/GG + Ceramics + nanoparticles)	^239,245,248,253^	High	High	Very high	Very high	Integrated functionality (mechanical + biological + angiogenic); biomimetic architectures; advanced performance	High complexity; reproducibility challenges; scalability issues

## 7 Challenges and Future Perspectives in Biopolymeric Bioinks for Bone Regeneration

Despite advances in biopolymeric bioinks for bone regeneration, some biological, mechanical, manufacturing, cost, and regulatory challenges need to be addressed.^260^ During the bioprinting process, a biological challenge is cell viability, as the exposure of cells to mechanical stresses, UV radiation, and shear forces can compromise cellular integrity.^261^ Furthermore, the integration of printed scaffolds with bone tissue is complex; biomaterials need to adapt to the environment, be biocompatible, avoid inflammation and immune rejection, and interact with the bone ECM to provide structural support and regulate cellular processes.^106^ Another biological challenge is related to controlling the rate of degradation and sustaining the cell growth. A bioink must gradually degrade at the same time as new bone tissue is being formed so as not to compromise the structure and function of the scaffold during the process. Scaffolds that degrade rapidly may lose their supporting function before the bone is regenerated, and scaffolds that degrade slowly may remain in the body for too long and interfere with natural bone remodeling.^163,262^

Mechanical challenges, optimizing the mechanical properties of bioinks, are very important for bioprinted scaffolds to adequately support the biomechanical forces of the bone environment.^151^ Generally, natural biopolymers (e.g., collagen, chitosan, cashew gum) have limited mechanical properties, making it necessary to modify these substances or combine them with synthetic polymers to ensure greater strength and rigidity.^115^ Another aspect to improve is the viscoelastic properties to ensure the printing of three-dimensional structures with high precision, porosity, and rigidity.^131^

In this context, the addition of nanoparticles to obtain composites can improve the mechanical properties and biocompatibility of the structure, with the aim of mimicking the structure and function of natural tissues. Nanocomposites can be designed to provide mechanical support, biocompatibility, and biological signals that promote tissue regeneration and repair. The use of nanoparticles, such as carbon nanotubes, graphene, and metallic nanoparticles, in biocompatible matrices, such as hydrogels, polymers, and ceramics, has been shown to improve the mechanical properties and biocompatibility of the resulting nanocomposites. Furthermore, these nanoparticles can be functionalized with biomolecules such as growth factors, peptides, and proteins to promote specific cellular responses and tissue regeneration. However, further research is needed to fully understand the long-term safety and efficacy of these materials in vivo.^263^

Regarding manufacturing, advances in smart bioinks and functionalization can be highlighted. Smart bioinks can respond to biological and physical stimuli, such as pH, temperature, or the presence of specific molecules, to optimize bone regeneration by releasing growth factors or modifying their properties in response to the local microenvironment.^264^ The functionalization of bioinks with osteoinductive growth factors (e.g., BMPs) and immunomodulatory agents can contribute, for example, to healing and bone tissue formation.^152^ Another challenge of the manufacturing process is the customization and scalability. Customizing scaffolds for each patient is one of the biggest advantages of 3D bioprinting, which can treat bone defects of different shapes and sizes.^265^

The main economic challenges are related to raw materials, manufacturing processes, infrastructure, scalability, and regulation. For this technology to be viable and applied in clinical medicine, it is necessary to overcome technical challenges and increase the scalability of bioink production, such as developing faster, more economical, and efficient bioprinting systems, together with the production of low-cost and easily accessible bioinks.^266,267^ Among the challenges related to formulation performance, the following can be mentioned: formulation stability, toxicity profiles, physiological interactions, immunogenicity, and manufacturing reproducibility, requiring robust preclinical validation, chemical and manufacturing controls, and postapproval pharmacovigilance.^263^

Regarding regulation,the transition from laboratory studies to clinical applications presents significant regulatory barriers. For exemple, bioinks and 3D bioprinted biomaterials must meet stringent safety, standardization, biocompatibility and efficacy requirements to ensure safe and effective treatment.^267,268^

In particular, the inherent batch-to-batch variability of natural materials can compromise the reproducibility, standardization, and product consistency. For instance, plant-derived biopolymers may vary due to geographic origin, seasonal factors, and climatic conditions, which can affect their quality and impact biomaterial fabrication and broader industrial applications.^269,270^

Because of their natural origin and high susceptibility to contamination, the sterilization of polysaccharides ensures that bioinks are free of microorganisms. However, this process may alter material properties such as structure, gelation, and rheological behavior, directly impacting the printability and performance of bioinks.^271^ Different methods, such as UV radiation, autoclaving, ethylene oxide, filtration, and lyophilization, have distinct effects on these properties.^274^ For example, in the case of alginate, sterile filtration followed by lyophilization showed better preservation of physicochemical properties and bioprinting performance, highlighting the importance of selecting an appropriate sterilization method.^272^

Through cross-linking, it is possible to improve the mechanical properties and stability of biopolymers in bioinks, enabling their application in tissue engineering and bioprinting.^273^ However, this process may compromise important natural properties, such as biodegradability and bioactivity. In addition, chemical cross-linking agents, particularly synthetic ones, may exhibit cytotoxicity, affecting cell viability and limiting clinical applications. In this context, there is growing interest in the use of natural cross-linking agents, such as genipin and citric acid, which offer lower toxicity and improved biocompatibility, although their performance still needs to be optimized to ensure adequate biomaterial properties.^274,275^ To achieve this, it is important to conduct clinical validation using animal and human models, which is typically a time-consuming and costly process and requires interdisciplinary collaboration among engineers, material scientists, and healthcare professionals.^153^

It is important to mention some products already approved by the FDA, such as Infuse, a bone graft containing recombinant human bone morphogenetic protein-2 (rhBMP-2) and an absorbable collagen sponge scaffold, and Euflexxa, which contains sodium hyaluronate and has been approved for the treatment of knee pain due to osteoarthritis. Currently, however, no synthetic hydrogel products have been approved for use in the regenerative market, presumably due to their low biocompatibility.^275^

In light of this, as future prospects, it is believed that continued innovation in 3D bioprinting technology, raw materials, and the design of new smart bioinks promises to overcome many of these challenges. The future of bioinks for bone regeneration includes the development of multifunctional materials to promote osteogenesis as well as soft tissue integration and vascularization, as well as optimizing the controlled release of drugs and growth factors.^14,163^ The combination of advanced bioinks with 4D, 5D, or 6D printing, which allows dynamic changes in material properties after printing, can further revolutionize the field, allowing scaffolds to be adapted over time according to the patient’s biological needs.^276,277^

The concept of 4D printing includes a temporal dimension that expands on the idea of 3D printing. Compared to 3D bioprinting, 4D bioprinting allows for the creation of dynamic, living constructs with adjustable properties that can accurately recapitulate the intrinsic dynamic and conformational changes of native tissues and potentially meet the need for dynamic, engineered tissues and organs. While there is no technological distinction between 3D and 4D printing, the main difference lies in the materials’ response to stimuli. 3D bioprinted products are typically static or undergo spontaneous natural changes without external stimuli. In contrast, 4D bioprinted products are expected to exhibit programmable and controllable dynamic changes triggered by physical, chemical, and biological stimuli (water, heat, pH, light, and electric and magnetic fields).^276–278^

5D printing is a process that involves the movement of the print head along five axes: X, Y, Z, rotation (A), and tilt (B), allowing for more complex and precise manipulations. The technique involves extruding 2D geometries in one direction, enabling the production of more complex structures and reducing printing time by minimizing vertical height during the printing process. While 6D printing technology emerges as an advanced form of additive manufacturing, integrating the principles of 4D and 5D printing to create objects capable of dynamic adaptation, this technique uses smart materials that respond to external stimuli, allowing 6D printing to create complex geometries with enhanced functionalities. This technology significantly reduces production times and material waste, becoming a more efficient and environmentally friendly alternative to traditional production methods, adding useful performance and energy efficiency to building models.^277,278^

## 8 Conclusion

Through the literature review, it is evident that the use of biopolymers in the formulation of bioinks for 3D bioprinting, specifically for bone regeneration, is a recent and emerging topic. Most studies have utilized the biopolymer alginate. Innovations are highlighted in various studies, such as the incorporation of nanocomposites, cells, bioactive compounds, and modifications in printing parameters. These advancements aim to achieve printable filaments, scaffolds with appropriate pore structures, and controlled cell release and biodegradation. Consequently, this review underscores the potential of employing other biopolymers in the formulation of bioinks for bone regeneration and other human tissues, as well as the benefits of combining biopolymers with other biomaterials.

## Data Availability

Data available on request due to privacy/ethical restrictions.
